# Familial Dysautonomia (FD) Human Embryonic Stem Cell Derived PNS Neurons Reveal that Synaptic Vesicular and Neuronal Transport Genes Are Directly or Indirectly Affected by *IKBKAP* Downregulation

**DOI:** 10.1371/journal.pone.0138807

**Published:** 2015-10-05

**Authors:** Sharon Lefler, Malkiel A. Cohen, Gal Kantor, David Cheishvili, Aviel Even, Anastasya Birger, Tikva Turetsky, Yaniv Gil, Sharona Even-Ram, Einat Aizenman, Nibal Bashir, Channa Maayan, Aharon Razin, Benjamim E. Reubinoff, Miguel Weil

**Affiliations:** 1 Laboratory for Neurodegenerative Diseases and Personalized Medicine, Department of Cell Research and Immunology, The George S. Wise Faculty of Life Sciences, The Sagol School of Neurosciences, Tel Aviv University, Tel Aviv, Israel; 2 The Hadassah Human Embryonic Stem Cell Research Center, The Goldyne Savad Institute of Gene Therapy, Hadassah Hebrew University Medical Center, Ein Kerem, Jerusalem, Israel; 3 Department of Developmental Biology and Cancer Research, Institute of Medical Research Israel-Canada, Hebrew University Medical School, Jerusalem, Israel; 4 Department of Obstetrics and Gynecology, Hadassah Hebrew University Medical Center, Ein Kerem, Jerusalem, Israel; 5 Department of Obstetric and Gynecology, Hadassah Hospital Mount Scopus, Hebrew University Medical School, Jerusalem, Israel; 6 Department of Pediatrics, Hadassah Hospital Mount Scopus, Hebrew University Medical School, Jerusalem, Israel; The University of Tennessee Health Science Center, UNITED STATES

## Abstract

A splicing mutation in the *IKBKAP* gene causes Familial Dysautonomia (FD), affecting the IKAP protein expression levels and proper development and function of the peripheral nervous system (PNS). Here we found new molecular insights for the IKAP role and the impact of the FD mutation in the human PNS lineage by using a novel and unique human embryonic stem cell (hESC) line homozygous to the FD mutation originated by pre implantation genetic diagnosis (PGD) analysis. We found that *IKBKAP* downregulation during PNS differentiation affects normal migration in FD-hESC derived neural crest cells (NCC) while at later stages the PNS neurons show reduced intracellular colocalization between vesicular proteins and IKAP. Comparative wide transcriptome analysis of FD and WT hESC-derived neurons together with the analysis of human brains from FD and WT 12 weeks old embryos and experimental validation of the results confirmed that synaptic vesicular and neuronal transport genes are directly or indirectly affected by *IKBKAP* downregulation in FD neurons. Moreover we show that kinetin (a drug that corrects *IKBKAP* alternative splicing) promotes the recovery of IKAP expression and these IKAP functional associated genes identified in the study. Altogether, these results support the view that IKAP might be a vesicular like protein that might be involved in neuronal transport in hESC derived PNS neurons. This function seems to be mostly affected in FD-hESC derived PNS neurons probably reflecting some PNS neuronal dysfunction observed in FD.

## Introduction

Familial Dysautonomia (FD; Riley-Day syndrome, hereditary sensory and autonomic neuropathy type III) is an autosomal recessive congenital neuropathy that affects the PNS [1, 2]. FD is caused by mutations in the *IKBKAP* gene, located in chromosome 9q31 [3, 4], which encodes a protein termed IkappaB kinase complex-associated protein or human Elongator factor1 (IKAP and hELP1 respectively). The major mutation (IVS20^+6T to C^) predominant in over 99 percent of FD patients of Jewish Ashkenazi origin, causes skipping of exon 20 and results in a frameshift that generates a truncated protein which seems to be unstable or undetectable [5]. *IKBKAP* mRNA from FD patients contains two isoforms, one in which exon 20 is present (normal), and the other in which exon 20 is spliced out (mutant) [6]. The mis-splicing has been shown to be under tissue-specific regulation and might also be regulated during development [5, 6]. The ratio between the two isoforms is variable and the abnormal splicing is shown to be predominant in the central and peripheral nervous systems [5]. The human *IKBKAP* gene contains 37 exons and encodes the 1332 amino acid (~150 KD) IKAP protein [7]. IKAP was found to be highly abundant and is considered to be expressed in most tissues [7]. Several cellular functions have been attributed to IKAP including as a scaffolding protein for the I-κB kinase complex (IKK) and therefore its name IKAP [2], as a member of the Transcription human elongator complex named human elongator protein-1 (hELP1) [8] or as a protein involved in stress response regulation of the c-jun N-terminal kinase (JNK)-signaling pathway [9]. Elongator complex subunits (ELP1/IKAP in particular) have been ascribed also to be required for modification of uridine residues in the wobble position of a subset of tRNAs in yeast [10–12], worms [13] and mammals [14]. Although existing evidence supports the cellular role for IKAP/hELP1 in the Elongator complex, the implication of the FD mutation in IKAP/hELP1 role in PNS development and the FD phenotype are far from being understood. Together with this it became clear that the knockout of *IKBKAP* or deletion of exon 20 in this gene is embryonic lethal in mice [15, 16]. Nevertheless, creation of a conditional *IKBKAP* transgenic mouse revealed the phenotype that recapitulates the major FD phenotypic and neuropathological features [17]. A recent study by George and colleagues [18] provides analysis of the cellular events that can go awry during sensory neurogenesis *in* a conditional *IKBKAP* knockout mouse model. In line with previous observations in chick embryos from Hunnicutt and colleagues [19], Jackson and colleagues [20] and ours [21], *IKBKAP* downregulation does not affect NCC migration, pathfinding, or DRG and sympathetic ganglia (SG) formation. Instead, *IKBKAP* appears to be essential for the second wave of neurogenesis of TrkA-positive nociceptors and thermoreceptors in the DRG [18] and in sympathetic and sensory target organ innervation [20, 21]. Alternatively, FD patient fibroblasts are an informative model of mRNA splicing regulation. However, our previous study suggests that IKAP/hELP1 expression is much higher in peripheral neurons derived from human embryonic stem cells (hESC) compared to primary fibroblasts [22], and fibroblasts do not exhibit the same ratio of *IKBKAP* exon 20 including: exon 20 skipping transcripts as observed in nervous system-derived tissues of FD patients [5]. This finding narrows the understanding of disease mechanisms in FD down to human cellular models where neural cells can be generated. Two examples of such FD models were produced either from induced pluripotent stem cell (hiPSC) derived from skin fibroblasts of FD patients [23] or derived from human olfactory ecto-mesenchymal stem cells (hOE-MSC) of FD patients [24]. Additionally, these two FD stem cell models were used for validating the potency of therapeutic agents such as kinetin, a cytokinin that has been shown to increase *IKBKAP* mRNA and protein expression in FD cell lines and *in vivo* models [25–27] as well as in leukocytes of healthy carriers of the FD mutation [28].

Human pluripotent stem cells provide new opportunities for authentic modeling of human diseases by deriving hESC from affected preimplantation genetic diagnosis (PGD) [29] embryos or developing induced hiPSC from patients [30]. To date, derivation of hESC lines from genetically abnormal embryos has been reported by few groups, including ours [31–37]. While affected hESCs were developed for substantial number of genetic disorders, disease phenotype was demonstrated only in one PGD hESC line [33], and therefore the usefulness of these lines as authentic disease models is yet to be further demonstrated. To study the role of IKAP/hELP1 in human neural crest cells and PNS neurons and to analyze the impact of the FD mutation in PNS neurons we developed a novel research cellular platform by deriving hESCs from an embryo that was found to be homozygous to the FD mutation in the course of PGD. Here we show that the FD-hESC has the potential to produce viable neural crest cells (NCC) and PNS neurons in spite of IKAP/hELP1 downregulation throughout the process. We further show that the FD-hESC derived NCC harbor a cell migration defect. Moreover the FD-hESC derived PNS neurons show a significant reduction in vesicular like colocalization with known neuronal vesicular proteins indicating potential defects in key neuronal functions such as synaptic vesicular and neuronal transport in this model. To strengthen this point we perform wide transcriptome analysis of FD and WT neurons and identified gene functional clusters and specific gene candidates within these specific neuronal functions affected in the FD cells. Pathophysiological confirmation to these results were obtained *in vivo* by comparing this data set with that obtained from the transcriptome analysis of human brains from FD and WT 12 weeks old embryos, indicating that the FD neurons undergo dysregulations of neuronal properties ranging from synaptic vesicle transport, ion gated channels and synapse organization. In validation experiments using either *IKBKAP* siRNA in WT hESC-derived neurons for direct *IKBKAP* downregulation or kinetin treatment in FD-hESC-derived neurons to restore *IKBKAP* levels we found that a set of candidate genes from these groups that show a similar expression pattern as *IKBKAP* in the FD background are either directly or indirectly affected by *IKBKAP* downregulation and most certainly functionally inter-connected via common pathways. Together with this we demonstrate that IKAP levels together with those of GRIA1 and Rab3a (selected from the validation experiments) can be restored in FD neurons after treatment with kinetin (known to overcome the FD mutation effect on alternative splicing of IKBKAP mRNA) suggesting that potentially neuronal function associated with these proteins is restored by kinetin treatment. Altogether, these results support the view that IKAP is probably a vesicular protein that is involved in specific neuronal functions related with synaptic integrity in human ESC derived PNS neurons. These functions seem to be mostly affected in FD-hESC derived PNS neurons probably reflecting some of the PNS dysfunction phenotypes observed in FD.

## Materials and Methods

### Ethics statements

#### Human embryos and PGD procedure

PGD embryos were donated by a couple undergoing IVF treatment at the IVF unit of the Hadassah Medical Center of The Hebrew University. Embryos that were not diagnosed as genetically normal were recruited for this study subject to signed written consent by the couples. The study was approved by the ethical committee at the Hadassah Medical Center as well as the Israeli Ministry of Health National Helsinki Committee for Genetic Research in Humans.

#### Human fetuses and tissue sample collection

Two human male 12 weeks fetuses were obtained from abortion procedures performed after approval of the hospital abortion committee. The study was approved by the ethical IRB of Hadassah Hebrew University Medical Center and the Israel Ministry of Health. The parents signed the inform consent for donation of tissues from the aborted fetuses for research.

### Differentiation of hESC to NCC

hESC colonies were collected using Collagenase IV, resuspended in CDM supplemented with SB431542 (10μM) and bFGF (20ng/ml), and cultured in non-adherent dishes. After 3 days, SB431542 was removed from the culture and clusters were further cultured in suspension in Chemically defined medium (CDM): DMEM/F12 (1:1), B27 supplementation (1:50), 2mM L-glutamine, 50U/ml penicillin, 50μg/ml streptomycin), supplemented with only bFGF. Day 7 clusters were then plated on fibronectin-coated (10μg/ml) culture dishes in CDM medium supplemented with bFGF and EGF (both 20ng/ml) for 24 hours to allow cell migration. The adherent clusters were then scraped off the plate, and the migratory NCC were cultured for further 24 hours in CDM with bFGF+EGF and formed a uniform cell population. NCC were passaged every 4–5 days with trypsin, up to 3 passages and were cultured on fibronectin-coated (10μg/ml) culture dishes in CDM with bFGF and EGF.

### Peripheral neuronal differentiation of hESCs

hESC colonies were collected using Collagenase IV. The free-floating hESC colonies were then collected, washed from the Collagenase IV by gravity and resuspended in differentiation medium. The hESC clumps were cultured in medium consisting of DMEM/F12 (1:1), B27 supplement (1:50), glutamine 2mM, 50 U/ml penicillin, 50 mg/ml streptomycin (Invitrogen, Carlsbad, CA), 20 ng/ml human r-bFGF (Peprotech, Asia), and 500–700 ng/ml human r-noggin (Peprotech, Asia) for 3 weeks, followed by removal of noggin. The formed NP spheres were propagated in the same medium that was refreshed every three to four days. The spheres size was maintained at ≤ 0.5mm in diameter by pipetting them through a fine tip. After noggin removal the hNPs were kept in suspension for several weeks in the presence of bFGF until attachment onto Poly D lysine, Laminin for terminal differentiation as we previously published [22].

### Confocal Immunofluorescence microscopy analysis

At the end of incubation, the cells grown on glass cover slips were gently rinsed in PBS and fixed with 4% paraformaldehyde/PBS for 20 minutes. After rinsing in PBS, the cover-slips were incubated for one hour in blocking solution containing 2% bovine serum albumin and 0.05% Triton in PBS. The following antibodies used in this study were diluted in blocking solution containing 2% FCS and 0.05% Triton x-100: Rabbit anti-Peripherin (1:200), Rabbit anti Gria1 (1:300) from Chemicon International, Temecula, CA, U.S.A.; Mouse monoclonal antibodies anti-SV2 (1:200), anti-hTAU (1:200), anti-Islet1 (1:200) from Developmental Studies Hybridoma Bank, Iowa, U.S.A.; Mouse anti-β-III-Tubulin ("TUJ1", 1:1000; Covance), rabbit anti-GFP (1:500, Invitrogen); Goat anti-Rab-3a (1: 300 Q-12 Santa Cruz Biotechnology 26552). Mouse anti-HNK-1 (1:100, a kind gift from Dr. Chaya Kalcheim, Hebrew University of Jerusalem). Mouse anti-human IKAP (1:300 BD Biosciences); Mouse anti-human IKAP (1:300 MO3, 6G9 Abnova Corporation) and Rabbit anti-human IKAP Antibodies (1: 300 H-302 Santa Cruz Biotechnology 8336) were used in this study. Secondary antibodies: Donkey anti-goat IgG-cy5, Donkey anti-mouse IgG-cy2, Goat anti-rabbit IgG-cy5 and Goat anti-mouse cy3 (1:1000; Jackson Laboratories ImmunoResearch). Cover slips were incubated with the primary antibodies for 1 hour at room temperature or overnight at 4°C. The secondary antibodies were applied for 1 hour of incubation at room temperature, together with Hoechst 33258 (0.1μg/ml) to stain cell nuclei. Cover slips were then rinsed in PBS, mounted on microscope slides in gel mount (BD Biosciences), and sealed with nail polish. Confocal analysis of the immnofluorescently labeled preparations was performed with LSM Meta confocal microscope (Carl Zeiss, Oberkochen, Germany). Image analysis was made using LSM software (Zeiss), Slidebook 4.0 (Intelligent Imaging Innovations, Inc), Photoshop (Adobe) and Imaris (Imaris, Bitpalne).

### Western blot analysis

Proteins were extracted from cell pellets using RIPA lysis buffer (Sigma-Aldrich Corp., Israel) Protein concentrations were checked using BCA kit (Pierce Biotechnology, IL, USA). For Western blot analysis, 30 mg proteins were loaded on 10% acrylamide gels or 4–15% gradient gels (Bio Rad) in Tris glycine buffer. Proteins were transferred to nitrocellulose membrane and blocked in 3% BSA (bovine serum albumin) in Tris buffer saline-Tween 20 (TBST) for 1 hr. Mouse monoclonal anti-hIKAP (BD Biosciences, Franklin Lakes, New Jersey, USA 1:500) or Rabbit anti-hIKAP (AnaSpec Inc. AS-54494 1:1000), mouse anti-β-actin ((Sigma-Aldrich Corp., Israel 1:1000), mouse anti-β-tubulin ((Sigma-Aldrich Corp., Israel 1:1000) were applied for 1 hr at room temperature. Secondary antibodies Donkey anti-mouse or Donkey anti-rabbit all HRP conjugated (at 1:10000) (Jackson ImmunoResearch laboratories, West Grove, PA, USA) were applied for 1 hr at room temperature. For enhanced chemiluminescence (ECL) autoradiography, Super signal kit (Pierce Biotechnology, IL, USA) was used following manufacturer instructions.

More information on materials and methods used in this work is available in S1 Supporting Information.

## Results

A human *in vitro* fertilized embryo that was diagnosed to be affected by FD was recruited for this study from our PGD program. A FD-hESC line was established from the laser-dissected ICM of the embryo, as previously described [35]. Details of the FD-hESC cell line characterization and genotyping are found in S1 Supporting Information and in S1–S3 Figs. *IKBKAP* mRNA expression analysis (S3 Fig) in WT-hESC and FD-hESC showed, as expected, preferential expression of the mutant alternative spliced *IKBKAP* mRNA in FD-hESC (excluding exon 20) as found in cells and tissues of FD patients, supporting its use for this study.

### Characterization of the PNS differentiation potential of the FD-hESC from NCC to neurons

**Fig 1:**
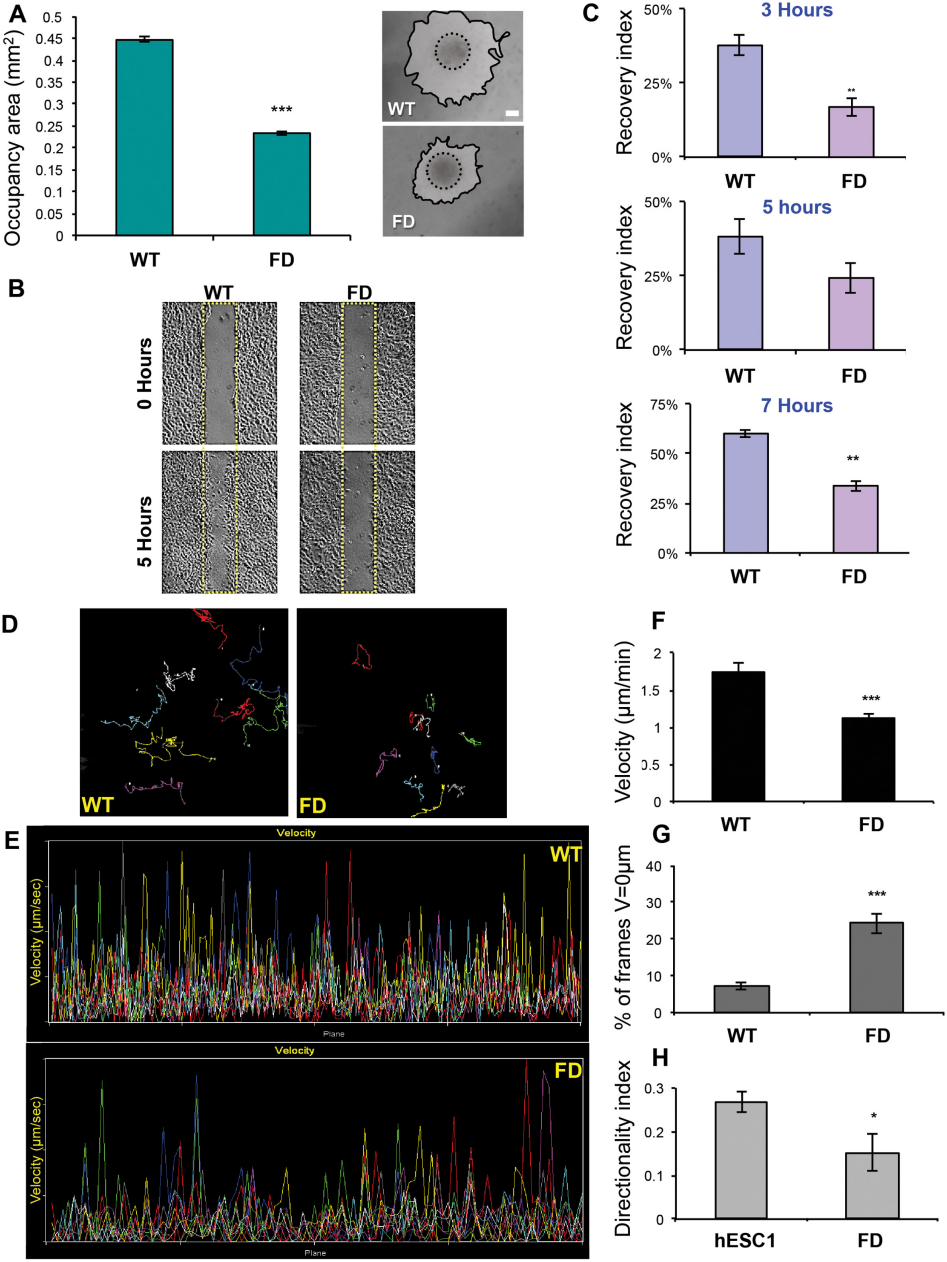
Analysis of FD-NCC migration. Delamination and migration of cells were monitored 24 hours after NP clusters attachment on Fibronectin. Migratory cells represent a homogenous population of NCC (See S4 Fig). Measurements of the areas occupied by the migrating NCC 24 hours after cluster attachment revealed that the FD-NCC had limited ability of delamination and migration. Representing phase contrast images of migration from plated clusters of WT and FD hNP clusters are presented on the right panel (A). Wound healing assay also showed reduced migration of FD-NCC. Representing phase contrast images of WT and FD-NCC cultures at time 0 and 5h after pipette tip scratching are shown in (B; Yellow rectangles represent original scratched area at time 0). FD-NCC have significantly reduced migration ability as indicated by their lower recovery index 3, 5 and 7 hours after scratching of the cultures (C). Time-lapse microscopy was later used to investigate FD-NCC migration in single cells level. The migration path of each WT and FD-NCC cell was traced and represented by different colored line, allow the follow-up of the migration path of each single cell (D). Monitoring of the velocity of each single cell is presented (each in specific color) on individually time frame during the assay (E). The measured mean migration velocity of FD-NCC is significantly reduced compared to WT-NCC (F). The mean percentage of time frames in which the FD-NCC were not migrating is significantly higher compared to WT-NCC (G). Finally, the measured directionality index of FD-NCC was significantly reduced as well (H). * P<0.05;** P<0.01; *** P<0.001; Scale bars A-B 200μm.

To study whether the FD mutation adversely affects early neural development, we characterized the early neural differentiation of the FD-hESC in comparison to WT-hESC. Neuralization was induced by culturing hESC clusters in suspension in the presence of Noggin and bFGF according to our published protocol [37]. Under these conditions, the FD-hESC underwent extensive neuralization similar to WT-hESC. After 4 weeks more than 90% of the cells in both FD and WT expressed the NPs marker PSA-NCAM (data not shown). We next characterized the differentiation of the FD hESC into homogenous NCC population, following an established protocol [38] as depicted in S4 Fig. While in these experiments we could not observe any differences in the efficiency of FD cells to differentiate to NCC, we have noticed that the delamination from plated human neural precursor (hNP) clusters and migration of FD-NCC was reduced in comparison to WT-NCC (Fig 1A). The area occupied by the NCC around the hNP clusters after 24 hours of migration was significantly smaller compared to WT-NCC. Further experiments to test the migratory capability of the FD-NCC were performed by using two methodologies: the wound healing assay and time lapse microscopy (see Fig 1B–1H). The wound healing assay tests the ability of the NCC to migrate over time and cover the gap surface made by scratch of a needle on a confluent NCC culture by photographing and measuring changes in the same gap areas for the period of 7 hours (Fig 1B and 1C). These results show that while WT-NCC succeeded to fill the gap with high recovery indexes, FD-NCC showed significantly lower recovery indexes at 3, 5 and 7 hours (Fig 1C). The time-lapse microscopy assay was used to investigate FD-NCC migration at the single cell level for up to 15 hours with a frame frequency of 1 every 5 minutes. Using the MetaMorph Microscopy Automation & Image Analysis software, we simultaneously tracked the migration path, frame by frame, of at least 10 cells per assay in WT and FD-NCC (Fig 1D). The velocities of the analyzed cells during their migration were determined (Fig 1E). The measured mean migration velocity of FD-NCC was significantly reduced compared to WT-NCC (Fig 1F) while the mean percentage of time frames in which the FD-NCC were not migrating was significantly higher compared to WT-NCC (Fig 1G). We further measured the time that the cells were committed to a certain direction and found that the directionality index of FD-NCC was significantly reduced (Fig 1H). Altogether these results demonstrate the reduced migratory capability of FD-hESC derived NCC.

We next further characterized the potential of the FD-hESC to differentiate into PNS neurons. To produce PNS-like neurons we followed the differentiation protocol as described previously [22]. Following 10 weeks of differentiation, cells had migrated from the hNP clusters and differentiated into cells with typical PNS-like neuronal morphologies. No obvious morphological differences were observed between WT and FD cell types throughout the 10 weeks of *in vitro* differentiation (see S5 Fig). The neuronal marker βIII-tubulin is expressed in 24 hours old neurites outgrowing from 8 weeks old WT and FD-hNP (see S5 Fig). The length of these outgrowing primary neurites at this stage was measured and no significant differences were found in neurite length nor in the number of neurites produced or of branching points counted along neurites between the two groups (data not shown), indicating that the initial outgrowth of neuronal projections is not affected in the FD cells. Moreover, the PNS differentiation lineage marker peripherin was detected in these hESC-derived WT and FD neuronal cells by immunofluorescence (see S5 Fig) indicating that the FD-hESC bear the potential to produce PNS neurons as WT-hESC.

**Fig 2:**
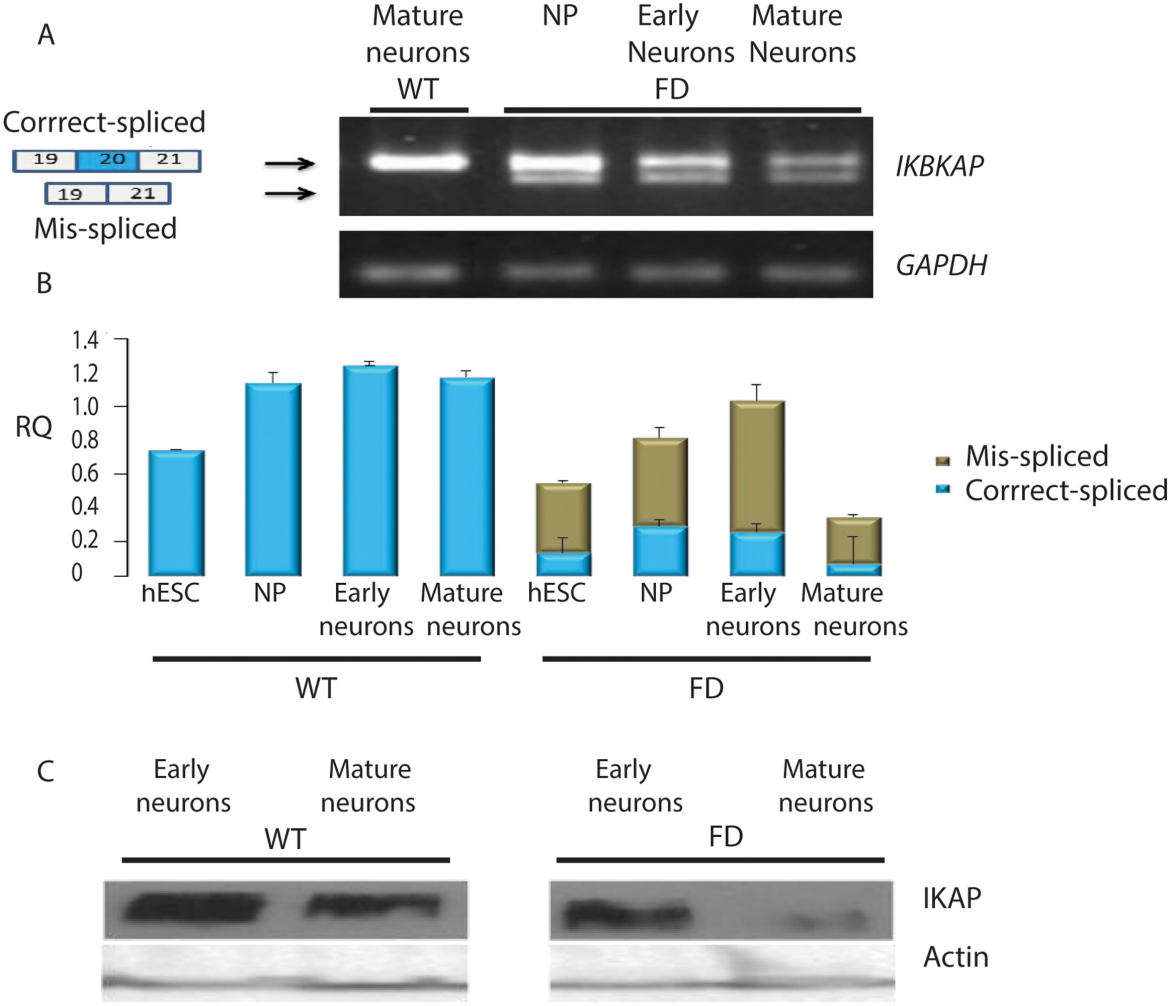
IKAP expression in WT and FD early and mature neurons. (A) RT-PCR analysis of the expression of *IKBKAP* showing WT (upper lane) and FD (mis-spliced, lower lane) mRNA isoforms at the stage of early neuronal precursors, early and mature neurons. (B) qRT-PCR analysis of the levels of *IKBKAP* WT (blue) and FD (green) mRNA isoforms. (C) Western blot analysis of IKAP protein levels in WT and FD early and mature neurons. Actin served as loading control.

**Fig 3:**
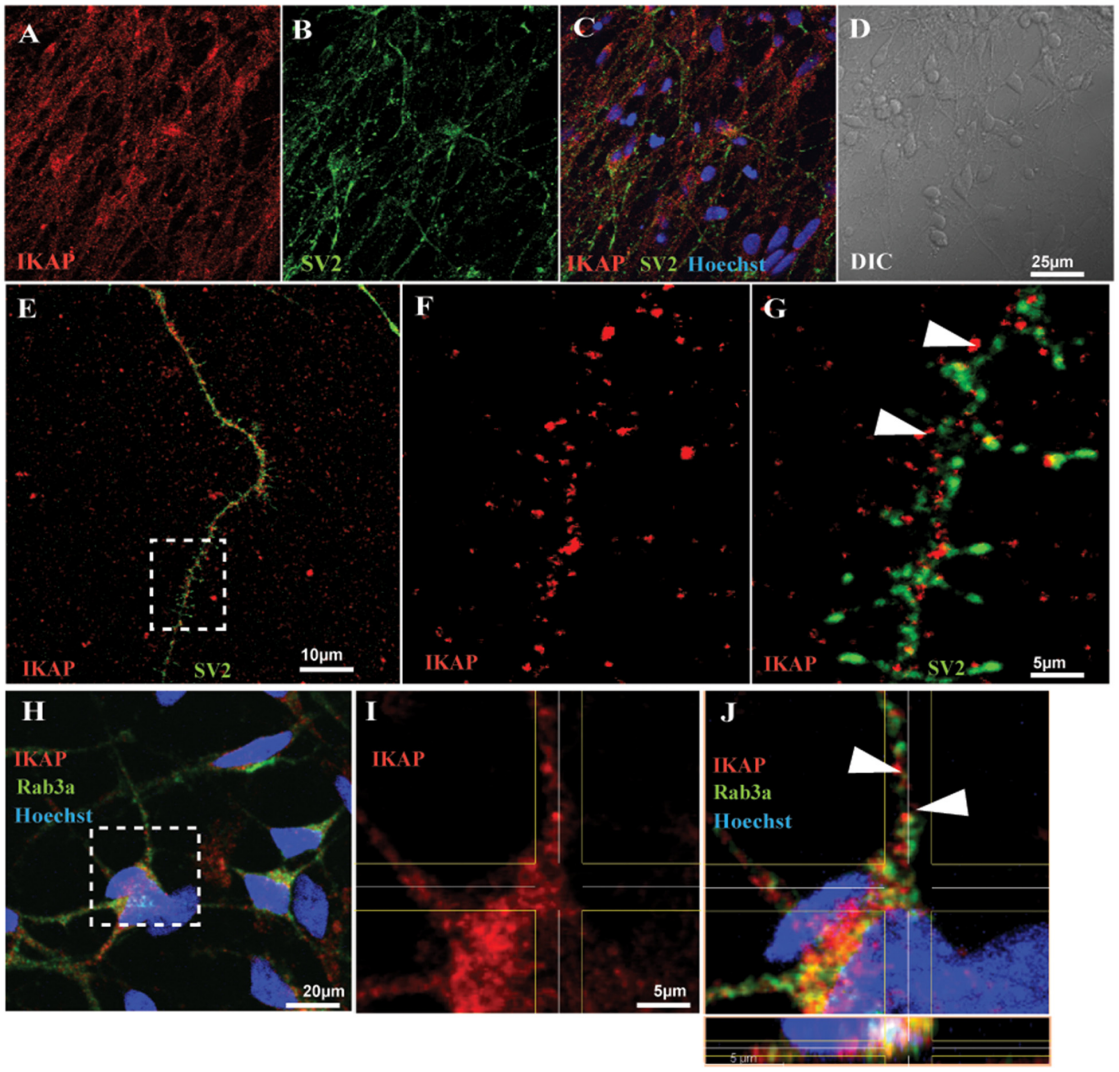
Localization of IKAP in hESC-derived PNS neurons in respect to vesicular neuronal markers SV2 and Rab3a. Confocal micrographs of hESC derived PNS neurons double-stained with Rabbit anti-hIKAP antibodies combined with either antibodies against synaptic vesicular markers, synaptic vesicle 2 (SV2) (A-G) or with Rab3a (H-J). (A-D) Low magnification images showing the IKAP and SV2 coexpression within neurites. (E-G) Magnification of a neurite exhibiting granular expression of IKAP and SV2. (F and G) High magnification of boxed area in E, showing partial colocalization of the two proteins (indicated by arrowheads in G) within distinct vesicle structures. (H) Low magnification image showing IKAP and Rab3a localization within the soma and along neurites. (I and J) High magnification of boxed area in H exhibiting partial protein colocalization pattern within distinct vesicles (indicated by arrowheads in J). Scale bars are indicated according to the magnification in each image.

**Fig 4:**
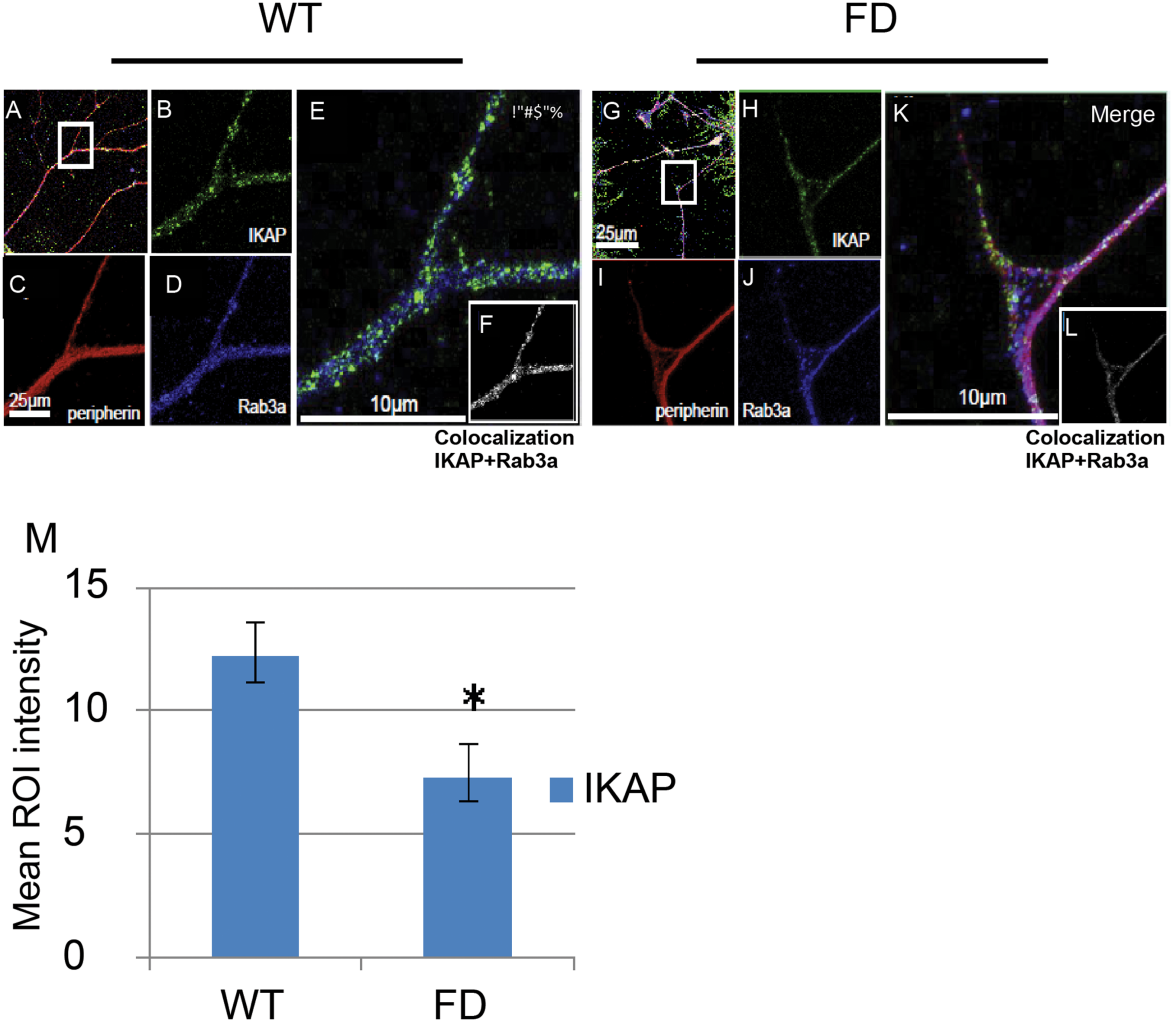
Characterization of IKAP localization in PNS WT and FD hESC-derived cultured neurons. Immunofluorescence confocal microscopy analysis was performed as shown in Fig 3. IKAP together with peripherin and Rab3a expression are shown within hESC derived neurons in WT (A-F) and FD (G-L) genetic backgrounds. Images B and H show the expression and localization of IKAP in WT and FD derived-neurons respectively. C and I, D and J show the expression of peripherin and Rab3a respectively. IKAP and Rab3a co-localization levels are shown in WT (F) and FD (L). Quantitative analysis of the mean intensity of IKAP and RAB3a colocalization levels in WT and FD axons is shown in M. 3D stacks of sequential confocal images were de-convolved using Huygens (scientific volume imaging-SVI) software and the analyzed for colocalization between the signals was performed using Imaris (Bitplan). Bar sizes are indicated in representative images. Mouse anti-hIKAP (BD Biosciences) was used in these experiments.

We next evaluated whether alternative splicing of the *IKBKAP* gene and the down regulation of the IKAP protein expression exist in the FD cells throughout the differentiation process. As a first step we compared the expression levels of the *IKBKAP* mRNA transcripts in FD and WT-hESC through the neuronal differentiation process. RNA was extracted at four time points along the PNS differentiation process; at time 0 (hESC pluripotent stage), at 6 weeks (hNP stage), at 8 weeks (early neurons stage) at 10 weeks, and after 2 weeks of plating on PDL/laminin (mature neurons stage) and semi-quantitative and quantitative RT-PCR analyses were performed as shown in Fig 2. Fig 2A shows the two mRNA transcript products of the FD mutated *IKBKAP*, typically observed in tissues of FD patients, are present throughout the PNS differentiation process. qRT-PCR analysis of *IKBKAP* expression in WT hESC-derived cells revealed higher levels as compared to the total *IKBKAP* transcript levels (including the FD-isoform) in FD cells at all stages of differentiation (Fig 2B). In the FD cells, *IKBKAP* expression levels were most dramatically down regulated in the mature neuronal stage. This was in line with the low IKAP protein levels detected by Western blot analysis in mature FD neurons as shown in Fig 2C. We further characterized the neuronal IKAP expression by immunofluorescence staining and confocal analysis. In wild type hESC-derived neurons IKAP expression appeared to be granular similar to the synaptic vesicles pattern commonly observed in neurons (see Fig 3). Double-staining with IKAP antibody and synaptic vesicle 2 antibody (SV2) confirmed such vesicular pattern (Fig 3A–3G). Together with this we show that IKAP and the synaptic vesicular marker Rab3a are partially colocalized as suggested by orthogonal image analysis (Fig 3H and in high magnification Fig 3I and 3J). In similar comparative experiments performed with WT and FD-hESC-derived neurons using Rab3a vesicular marker (see Fig 4), a reduction in IKAP/Rab-3a vesicular staining colocalization can be observed comparing WT and FD cells (Fig 4F and 4L respectively). This reduction in IKAP staining associated with Rab3a labeled vesicles in the FD cells was confirmed by quantitative analysis of these images (Fig 4M). Altogether these results show that IKAP might be associated with vesicles suggesting a possible role in neuronal transport that might be affected in FD neurons.

**Fig 5:**
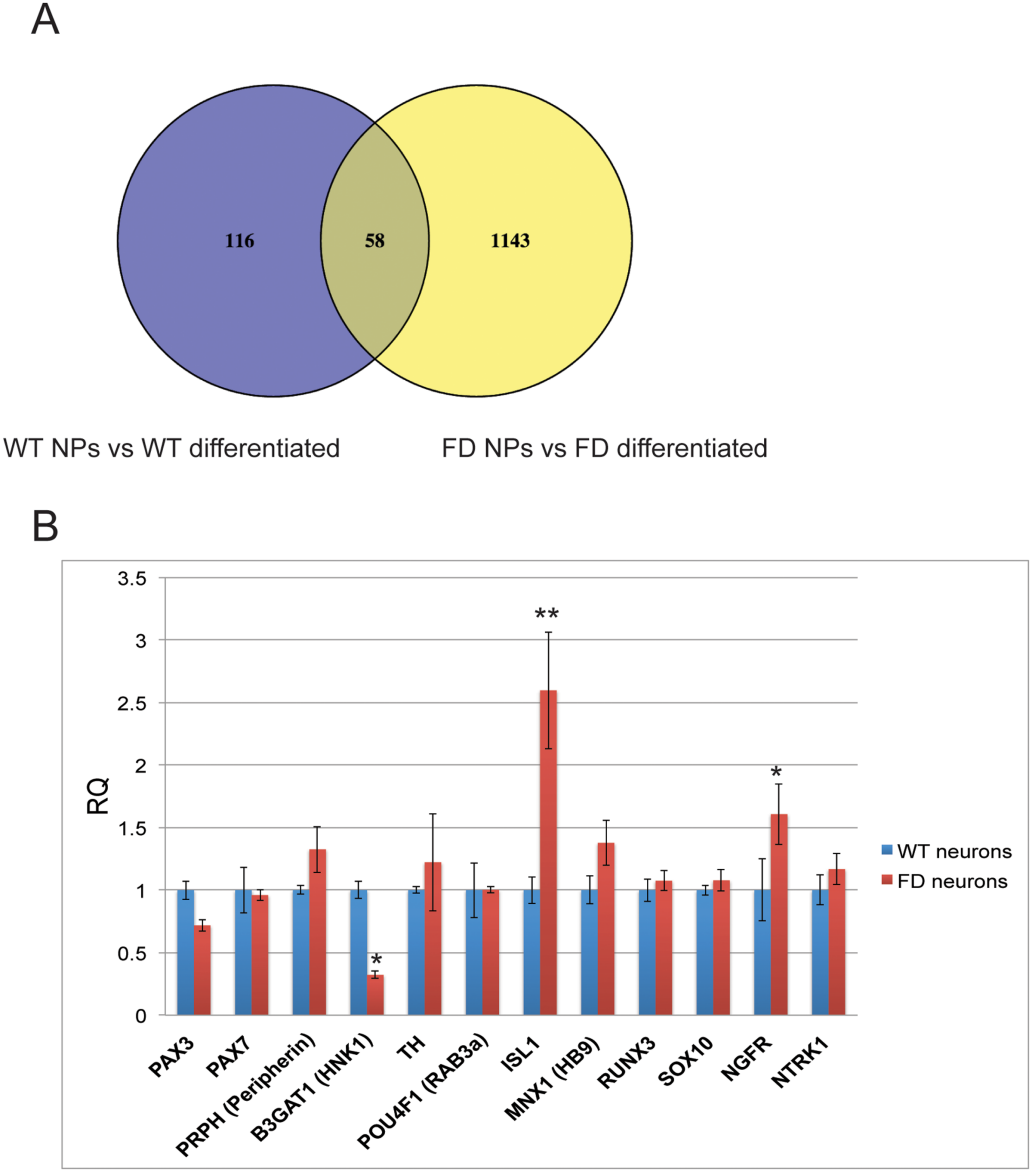
Comparative analysis of expression differences in WT and FD hESC derived PNS differentiation process. (A) Venn diagram comparing the overall number of genes that showed > 2 fold expression differences between hNP and fully differentiated hESC-derived neurons in WT (in blue) and FD (in yellow) backgrounds. (B) The normalized relative quantification expression values are shown as mean ± s.d. of several developmental PNS markers and transcription factors in WT and FD hESC-derived neurons as obtained in the transcriptome cDNA chip analysis. * P<0.05 **P<0.01.

To address this possibility we performed a gene expression transcriptome profiling to study the genome wide effect of the FD mutation in the *IKBKAP* gene during PNS neuronal differentiation. Total mRNA was extracted from FD and WT hESC-derived neurons at two differentiation stages: early, 6 weeks NPs; and late, 9 weeks mature neurons. The mRNA samples from 3 independent experiments was reverse-transcribed and applied onto an Affymetrix 32k human cDNA chip array in triplicates, and expression levels of all transcripts in the different samples were measured. Analysis of the global gene expression pattern in the samples show distinct differences between differentiated FD and WT neurons that are manifested at the time of terminal differentiation. Fig 5 shows the results from this analysis. This analysis showed as expected that *IKBKAP* expression levels were significantly lower than in the respective WT samples (data not shown). The number of target genes that are significantly altered in their expression following neuronal differentiation in WT (174 genes) and in FD (1201 genes) are represented in Fig 5A. Having found that the major differences in gene expression between WT and FD hESC are manifested at the final stage of neuronal differentiation we focused our further studies at this time point. To understand the effect of IKAP deficiency on PNS differentiation in FD neurons, we first analyzed the expression levels of different known developmental PNS-markers in FD and WT samples as done previously [22, 39]. Fig 5B shows the normalized values of this analysis, indicating a significant down regulation in HNK1, a protein related to migrating neural crest population and PNS precursors derivatives [40]. In addition, the PNS neuronal markers, Pax-3, Pax-7, peripherin, tyrosine hydroxylase (TH), Sox-10, Runx-3, NGFR (p-75) and NTRK1 did not show a significant difference in their expression in the FD neurons as compared to WT. In contrast, the nerve growth factor receptor NGFR and Islet-1 were up regulated significantly; the latter, is a transcription factor known as a marker of dorsal root ganglia (DRG) pools of PNS neurons as well as motor neuron pools at the ventral spinal cord in the CNS during neural development [41]. However the transcription level of the motor neuron marker HB9 was not found to be elevated in the same manner, suggesting that the effect was probably related specifically to the Islet-1 role in PNS lineage. Altogether these results strongly support the PNS lineage of the FD-derived neurons.

### IKAP deficiency affects gene expression of neuronal functional genes in FD hESC-derived neurons

The effect of IKAP deficiency on neuronal properties was examined in the transcriptome wide analysis using gene ontology (GO) data analysis. This analysis allows us to understand which pathways and functions have been altered in FD neurons due to low IKAP levels. We used GO analysis on gene expression readings from WT and FD fully-differentiated neurons, in which *IKBKAP* levels showed the highest variance. Genes that had at least 2 fold differences between WT and FD were selected. The processes and functions attributed to genes that are downregulated (1201 genes) and upregulated (522 genes) in FD neurons by GO analysis are summarized in S1 Table. Within the group of downregulated genes it appears that the majority of genes affected in FD neurons are related to neuronal growth and function. From these results we conclude that molecular aspects regarding neuronal migration, outgrowth, guidance, synapse assembly, and cytoskeleton organization are mostly affected in FD neurons. The affected processes appear to be caused by changes in genes involved in the function of actin and clathrin binding, calcium/calmodulin binding, GTPase activity, and intermediate filament binding. S1 Table also shows significant changes in gene clusters involved in membrane depolarization and transmission of nerve impulses reflected by changes in GABA and glutamate receptors function as well as GTPase and ATPase activity. In contrast GO analysis of upregulated genes in FD neurons shows that the main effect of IKAP deficiency is on genes that are involved in morphogenesis in general. These changes are in genes involved in extracellular matrix binding that are present in a broad spectrum of cell types including neurons. Interestingly, while the downregulation effects seems to be mostly neural specific, the upregulation effects on gene expression detected in FD neurons are mostly genes involved in non-neuronal specific processes, that are expressed also in other tissues such as skeletal muscles or kidney development. Details of the affected genes from the clusters described in S1 Table are shown in S2 Table.

### Physiological relevance of hESC-FD-derived neurons as a model

**Fig 6:**
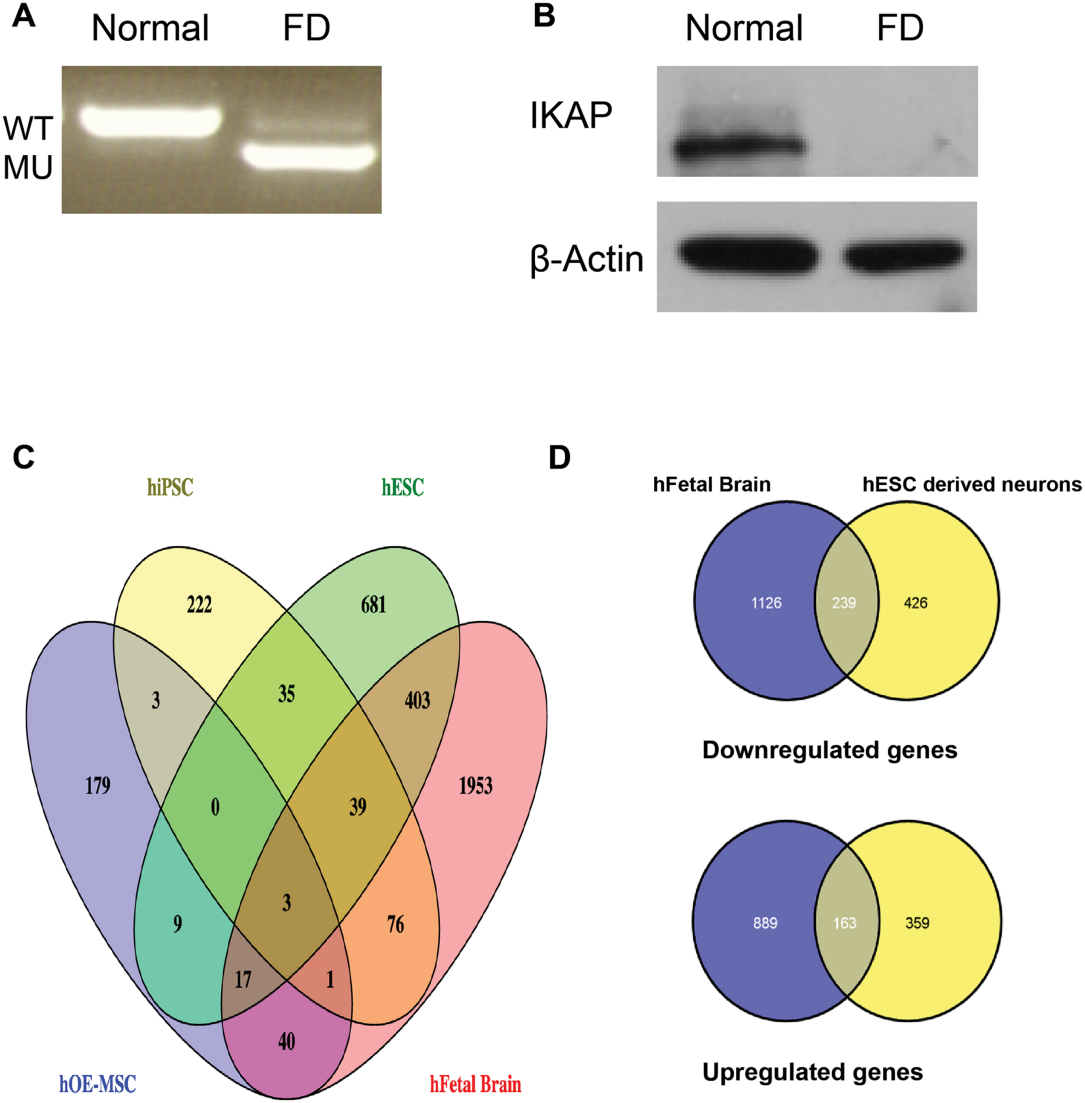
Comparative transcriptome analysis between human 12 weeks fetal WT and FD brains to WT and FD hESC derived neurons. Prior to cDNA microarray chip analysis mRNA and protein extracts from the brain samples were used to validate the FD phenotype of the FD splicing mutation in the *IKBKAP* gene at the transcriptional and translational levels by: (A) RT-PCR analysis of the expression of *IKBKAP* in human 12 weeks fetal WT and FD brains showing WT (upper lane) and FD (mis-spliced, lower lane) mRNA isoforms in the FD brain only. (B) Western blot analysis of IKAP protein levels in WT and FD brains. Note the almost complete absence of IKAP in the FD brain. β-Actin served as loading control. Comparative transcriptome analysis between data obtained from both cDNA microarray chips of WT and FD fetal brains and hESC-derived PNS neurons was performed by cross-referencing genes which their expression levels differ significantly (>2 fold) between FD and WT. (C) Venn Diagram representation of a wide genome transcriptome analysis of common genes that are differentially expressed in FD-hESC-derived PNS neurons (in green) and in two other known FD stem cell neural-derived models (FD fibroblasts derived iPSC (in yellow) [23] and FD-hOE-MSC (in blue) [24] and in FD Fetal brain (in red). Cross-referencing genes with their expression levels difference of >2 fold change for hESC p<0.05 or >1.5fold change p<0.05 for iPSC and hOE-MSC between WT and FD were considered for analysis. The number at the crossection between the diagrams represents the number of genes that are shared by these multiple analyses. For the list of genes see S3 Table. (D) Gene sets were divided into downregulated and upregulated genes in both hESC-derived PNS neurons and Fetal brain biological systems. Venn diagram represents the results from this analysis showing in blue, the number of genes that differ in WT and FD in the fetal *in vivo* and in yellow, the number of genes that differ in WT and FD hESC derived neurons *in vitro*. The crossection between the diagrams represent the number of genes that are shared by both analyses. Upper diagrams represent the downregulated genes while the lower ones represent the upregulated genes.

To investigate the pathophysiological relevance of our microarray results of hESC WT and FD-derived PNS neuronal cells in perspective with the available microarray data analysis (kindly provided by Dr. EC Ibrahim, Marseille University as described by Boone and colleagues [42] of two established FD stem cell models at their most advanced neural differentiation stage (FD fibroblasts derived iPSC [23] and FD-hOE-MSC [24], we compared these data with those obtained from an *in vivo* cDNA microarray chip made from FD and WT 12-week-old male fetal brains. The analysis was performed by cross-referencing genes of which their expression levels differ significantly (>2 fold change and a *P*-value < 0.05 for hESC or >1.5 fold change and a *P*-value < 0.05 for iPSC and hOE-MSC [42] between WT and FD *in vitro* with those found in the brains of 12 weeks WT and FD embryos as depicted in Fig 6. To validate this *in vivo* FD model we performed RT-PCR and Western blot analyses of *IKBKAP* expression showing the FD-splicing mRNA expression phenotype as well as the expected *IKBKAP* downregulation in the FD-brain (Fig 6A and 6B). A Venn Diagram representation of a genome-wide transcriptome analysis of common genes that are differentially expressed in the three FD stem cell models mentioned above and FD Fetal brain is shown in Fig 6C. This comparative analysis was performed in a similar manner as previously described [42] combining our differential gene expression data from our FD in vitro and in vivo models with the previously analyzed data of the FD hiPSC and hOE-MSC models. Interestingly, the FD fetal brain showed the highest total number of genes (2532) that are expressed differentially from the WT sample followed by the FD-hESC-derived PNS neurons with a total of 1187 genes, the neural differentiated hiPSC with 379 and the hOE-MSC with 252 genes differentially expressed. Moreover, the amount of overlapping genes between each of the three FD stem cell neural models with the FD brain sample is 462, 119 and 61 for the hESC, hiPSC and hOE-MSC respectively (see details of the genes from this analysis in S3 Table). Surprisingly, no overlap genes were found in between the three FD stem cell models while only 3 genes were found in the cross section between them and the FD fetal brain. *IKBKAP* was found in the cross section between our FD models and hOE-MSC. These results clearly indicate that the FD-hESC-derived PNS neuron *in vitro* model best reflects the gene expression changes detected in the FD fetal brain in vivo. To study in more depth the relevance of the overlapping genes between the FD-hESC-derived PNS neurons and FD fetal brain samples gene sets were divided into downregulated and upregulated genes in both biological systems. Venn diagram analysis of the results show that about 30% of the genes in the FD neurons *in vitro* in both sets of downregulated (239 out of 665) and upregulated (163 out of 522) genes also exhibit a similar pattern in FD fetal brains (Fig 6D). In addition, the comparison between the two different neuronal FD models helps to narrow down the background genes, which are non-neuronal IKAP deficiency related. It should be noted that the chip array used for the *in vivo* material was of 44k probes set in comparison to the 32k Affimetrix used for the *in vitro* data set, increasing the total number of differentiated *in vivo* genes in the analysis. Differential genes resulting from these cross-references were categorized according to the GO annotation database. Table 1 summarizes the processes (A) and functions (B) mostly affected by downregulated and upregulated genes found in this analysis. The downregulated genes that are mostly affected in FD neurons and FD brains are related to the synaptic integrity process including genes involved in synaptic transport, synaptogenesis, and synapse maintenance, as well as neuronal ion channel (in particular glutamate, AMPA receptors) and GTPase activity (Table 1A). This can, in turn, functionally affect the synaptic property, neuronal plasticity, and neuronal cell survival (Table 1B). In contrast the upregulated GO enrichments results are related to general cellular processes, such as morphogenesis, cell proliferation and cell adhesion, which affect several cell signaling pathways such as Wnt pathway via adhesion molecules, calcium binding and extracellular matrix in Table 1B. Details of the affected genes from the clusters described in Table 1 are shown in S4 Table. The above results show that the FD neurons undergo dysregulations of neuronal properties ranging from synaptic vesicle transport, ion gated channels and synapse organization. Genes that are highly represented in synaptic transmission in FD neurons and FD brains were found to be ion-gated channels specific for neuron pre and post-synaptic excitability (see S5 Table). These finding may indicate a major defect in excitability properties in FD neurons *in vitro* which could possibly reflect PNS neuronal dysfunction in FD.

**Table 1:** GO analysis of differential gene expression between hESC-derived PNS neurons and embryo brains of WT and FD origins. The major enriched GO analysis groups showing processes (A) and gene functions (B) from the two in vivo and in vitro cDNA microarray chip data. The enrichment groups are arranged by number of downregulated and upregulated genes detected in each group. Only genes above 2-fold change are included. FDR q-value (adjusted p-values of result significance), Enrichment = (number intersection genes / input genes) / (total number of genes of specific GO term / background genes).

**A**			
**Process description**	**FDR q-value**	**Enrichment**	**Gene number**
**Downregulated genes**			
cytoskeleton organization	2.99E-02	1.86	36
axon guidance	3.68E-05	2.79	34
nervous system development	1.17E-06	3.44	33
positive regulation of GTPase activity	4.20E-03	3.22	17
neuron migration	9.39E-05	4.66	16
neuron projection development	4.89E-02	3.27	11
membrane depolarization	2.32E-02	3.98	10
regulation of dendrite development	4.26E-02	3.91	9
neuron-neuron synaptic transmission	9.67E-03	5.65	8
synapse assembly	2.08E-02	5.02	8
filopodium assembly	2.93E-03	8.24	7
regulation of neuronal synaptic plasticity	4.00E-02	5.07	7
behavioral response to pain	5.50E-02	9.41	4
regulation of neuron migration	4.25E-02	16.94	3
**Upregulated genes**			
cell adhesion	1.30E-04	4.87	15
embryonic limb morphogenesis	5.95E-07	26.34	9
regulation of cell migration	8.51E-03	5.48	9
tube formation	1.16E-02	12.25	5
collagen fibril organization	1.75E-04	32.44	5
regulation of skeletal muscle fiber development	9.08E-03	20.87	4
embryonic skeletal joint morphogenesis	4.60E-03	55.39	3
**B**			
**Function description**	**FDR q-value**	**Enrichment**	**Gene number**
**Downregulated genes**			
calcium ion binding	2.21E-05	2.39	52
actin binding	6.23E-02	2.09	26
GTPase activator activity	1.16E-01	2.23	19
calmodulin binding	1.61E-02	3.16	18
extracellular-glutamate-gated ion channel activity	6.57E-02	7.43	5
GABA-A receptor activity	6.85E-02	7.43	5
clathrin binding	6.24E-02	7.84	5
AMPA selective glutamate receptor activity	4.48E-02	21.18	3
benzodiazepine receptor activity	1.06E-01	14.12	3
intermediate filament binding	1.03E-01	14.12	3
**Upregulated genes**			
extracellular matrix structural constituent	2.86E-03	18.95	6
collagen binding	2.28E-03	26.67	5
calcium ion binding	2.48E-02	4.29	11
sulfur compound binding	7.84E-03	10.06	7
heparin binding	9.99E-03	11.25	6

**Fig 7:**
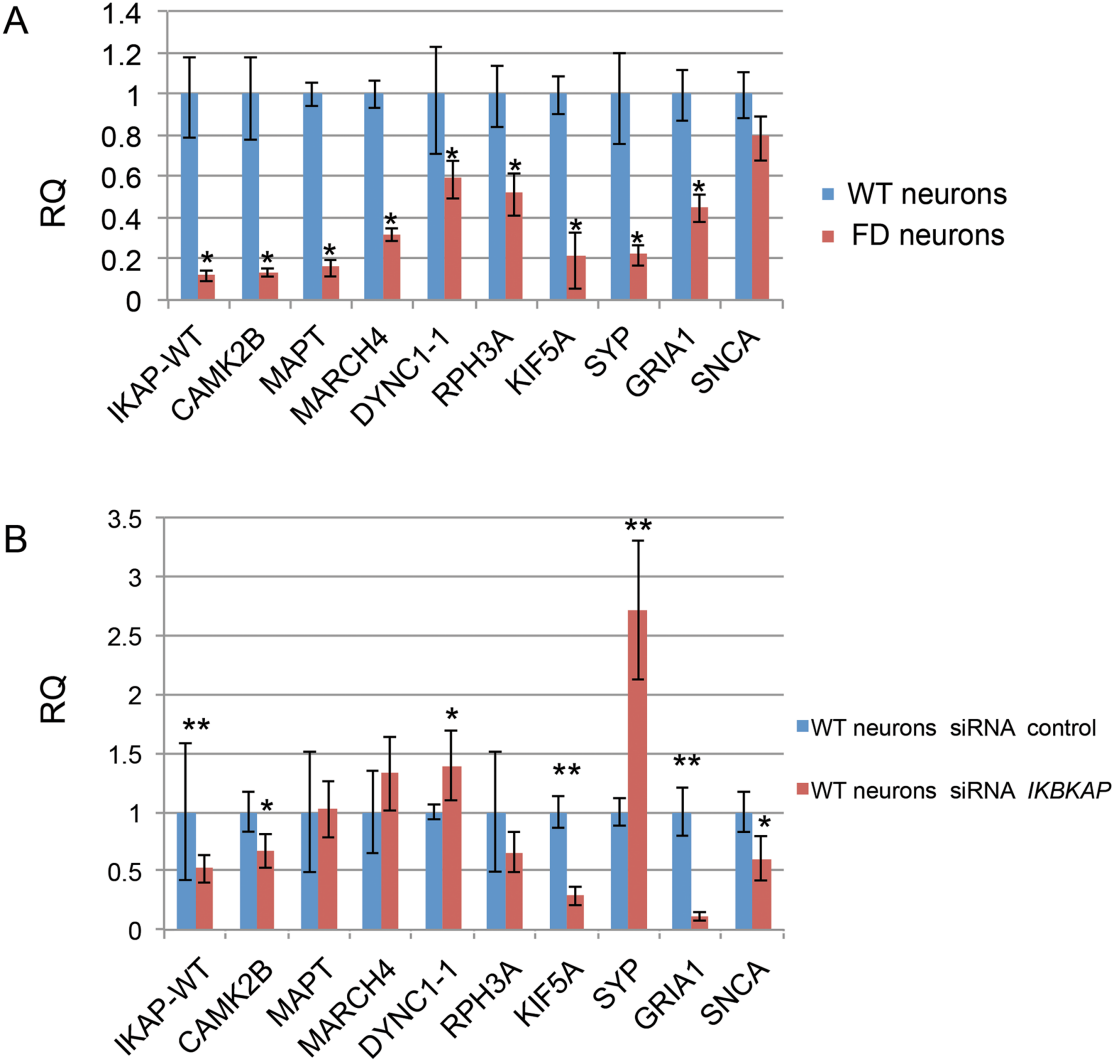
qRT-PCR analysis for validation of *IKBKAP* predicted co-regulated functional candidate genes in FD neurons and in siRNA *IKBKAP* downregulated WT neurons. Validation experiments for *IKBKAP* predicted co-regulated functional candidate genes selected using the “Expander” micro arrays analysis tool were performed by qRT-PCR analysis on cDNA produced from FD hESC derived PNS neurons and on siRNA *IKBKAP* downregulated WT hESC derived PNS neurons (A) qRT-PCR results of relative quantification levels of *IKBKAP* and “candidate genes” in WT vs FD hESC derived PNS neurons. (B) qRT-PCR results of relative quantification levels of *IKBKAP* and “candidate genes” in WT-hESC derived PNS neurons that were treated with control siRNA and siRNA *IKBKAP* represented as mean ± s.d. * P<0.05 **P<0.01, Non statistical significance (NS).

**Fig 8:**
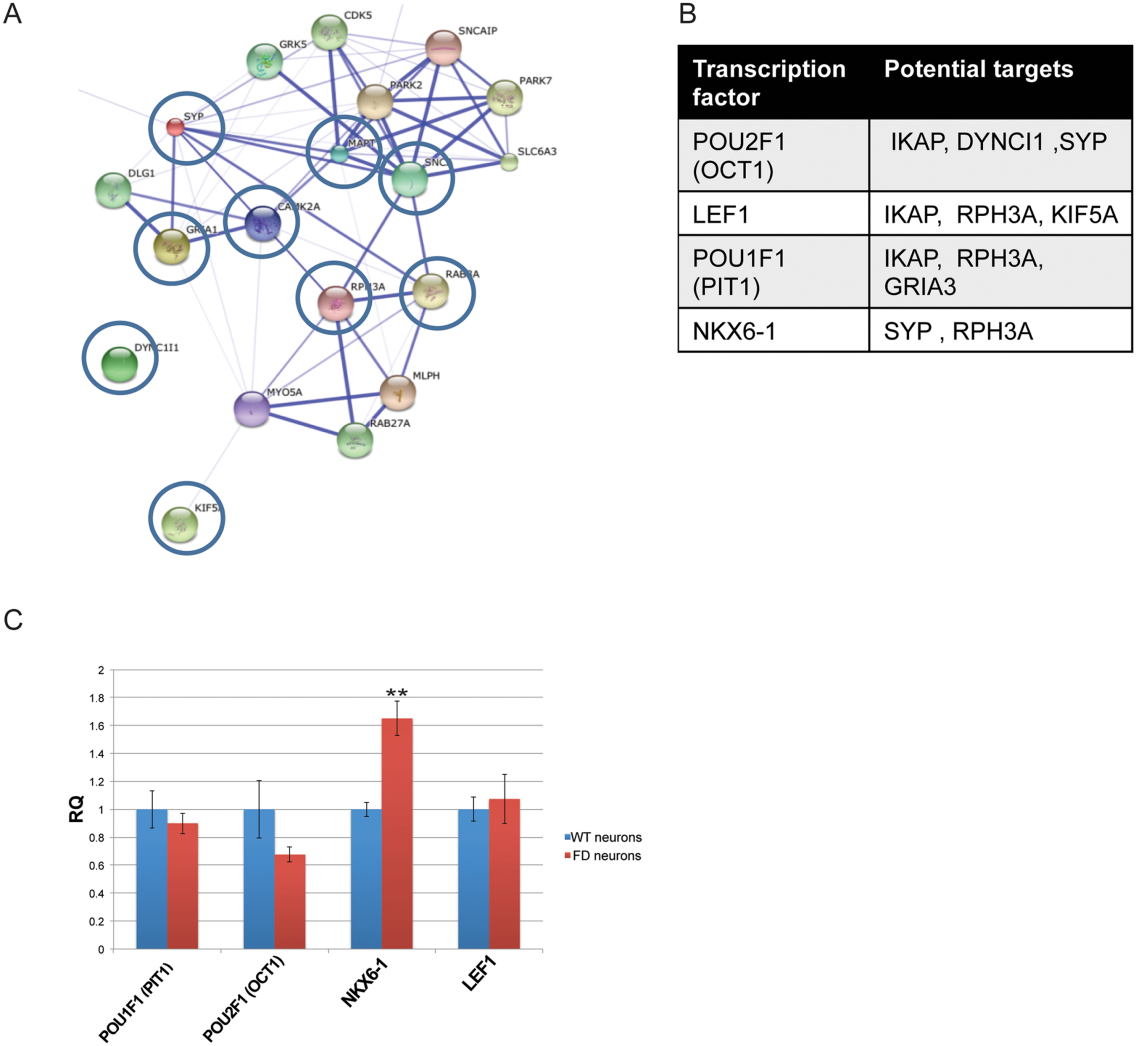
Prediction analysis of networks and transcription factors regulating *IKBKAP* and *IKBKAP* co-regulated functional candidate genes. “String” analysis of protein networks showing the potential interaction between the majority of the *IKBKAP* co-regulated functional candidate genes. (B) Prediction of transcription factors (TF) related to *IKBKAP* and co-regulated functional candidate genes. (C) Shows relative quantification levels represented as mean ± s.d. of TF gene candidates taken from the total cDNA microarray analysis showing difference between WT and FD-hESC derived PNS neurons. * P<0.05 **P<0.01, Non statistical significance (NS).

Following the cDNA microarray analysis where we found a clear effect of *IKBKAP* downregulation on genes involved in neuronal activity in FD neurons *in vitro* and *in vivo*, we selected several genes that show a similar expression pattern as *IKBKAP*, using the “Expander” micro arrays analysis tool. These sets of genes may be co-regulated with the *IKBKAP* gene and/or related to the IKAP function and either directly or indirectly affected by *IKBKAP* downregulation. The selected genes belong to the following functional categories listed in Table 1: (i) Transport machinery; dynein, cytoplasmic 1, intermediate chain 1 (DYNC1I1), kinesin family member 5A (KIF5A) (ii) Neuronal activity and exocytosis; Synaptophysin (SYP), glutamate receptor, ionotropic, AMPA 1 (GRIA1). (iii) Signal transduction; calcium/calmodulin-dependent protein kinase II alpha (CAMK2A), Rabphilin 3A homolog (RPH3A). (iv) Ubiquitin associated; membrane-associated ring finger (C3HC4) 4, E3 ubiquitin protein ligase (MARCH4). (v) Cytoskeletal; microtubule-associated protein tau (MAPT). (vi) Neurodegenerative associated; synuclein, alpha (SNCA). To validate these results, qRT-PCR analysis of these genes was performed on new samples of WT and FD-hESC-derived neurons as shown in Fig 7A. Most selected genes showed significant lower expression levels in FD in comparison to WT neurons with the exception of SNCA that showed no significance. To investigate the possibility that *IKBKAP* downregulation affects specifically the expression of the selected genes in FD neurons we induced *IKBKAP* downregulation by transfection of human *IKBKAP* siRNA or GFP siRNA (as negative control) of fully differentiated hESC-derived neurons of WT origin. Fig 7B shows the results of these experiments where IKBKAP level was downregulated by approximately 50% following 48 hours of siRNA transfection. This abrupt reduction in *IKBKAP* expression mainly affected the expression levels of CAMK2B, RPH3A, KIF5A, SNCA and, most prominently, GRIA1. In contrast, an elevation in the mRNA levels of MARCH4, DYNC1A and, SYP was observed in these experiments. This and other existing discrepancies in the *IKBKAP* downregulation effect on certain genes could be attributed to the fact that FD cells were exposed to constitutive low *IKBKAP* expression levels throughout the differentiation process starting at the early pluripotency stage, while the effect induced by *IKBKAP* siRNA is an immediate effect of *IKBKAP* deficiency in the system. Three of the most downregulated genes in both *IKBKAP* downregulation systems were GRIA1, KIF5A and RPH3A which are known to be involved, via the GRIP adaptor protein, in regulating and assembly of glutamate AMPA receptors in neurons [43]. Altogether these results indicate the possibility that a potential co-regulation effect is caused by IKAP deficiency on the above selected genes. To investigate this possibility we analyzed the known molecular interaction and the potential transcription factors (TF) that might be involved in their expression regulation using bioinformatics tools. Fig 8A shows the results obtained from “string” analysis, which identifies known and potential protein interactions of the candidate genes (circled in blue) identified above. Interestingly, String analysis shows first and second degree associations for the majority of the selected genes. This may indicate that these genes are functionally inter-connected via common pathways. In addition, potential TF binding sites on few of these selected genes were identified using “PRIMA” software via the “Expander” analysis tool (Fig 8B). Four TF were identified by this bioinformatics tool, in which OCT1, LEF1, and PIT1 were found to potentially regulate *IKBKAP* itself, strongly indicating a transcriptional co-regulation between *IKBKAP* and some of the selected target genes. Interestingly, preliminary evidence to support this assumption was found in the cDNA transcriptome analysis described above showing that NKX6.1 from these four TF is expressed significantly different in hESC-FD neurons as compared with control (Fig 8C).

### Effect of kinetin treatment on IKAP candidate gene targets in FD hESC-derived neurons

**Fig 9:**
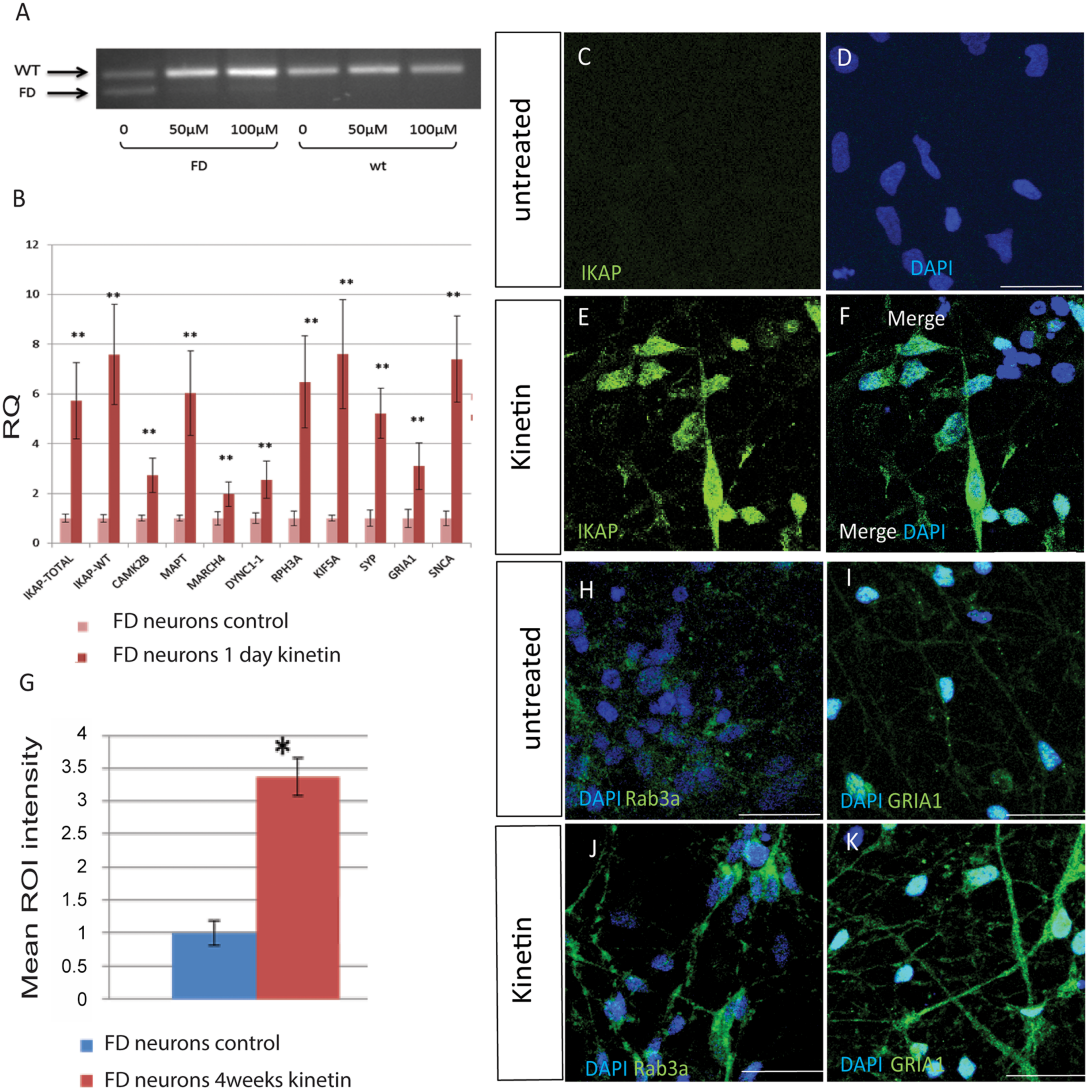
Rescue effect of kinetin on *IKBKAP* expression and concomitantly on in FD-hESC derived PNS neurons. (A) RT-PCR results of one day kinetin treatment on FD-hESC derived PNS neurons showing the drug correcting effect on FD alternative splicing. (B) qRT-PCR analysis of the relative quantification levels represented as mean ± s.d. of *IKBKAP* and co-regulated functional candidates in FD neurons following one day kinetin treatment. * P<0.05 **P<0.01. (C-K) Effect of 4 weeks kinetin treatment on the expression of IKAP in FD-hESC derived PNS neurons (C-D, untreated and E-F, 4 weeks kinetin treated by immunofluorescence confocal analysis. (G) Quantification of IKAP mean levels calculated from mean RGB after 4 weeks kinetin treatment on these cells (C-K). * P<0.05. (H-K) Effect of 4 weeks kinetin treatment (J and K) on the expression of Rab3a (H and J) and GRIA1 (I and K) in FD-hESC derived PNS neurons by immunofluorescence confocal analysis. Mouse anti-hIKAP antibody (Abnova Corporation) was used in these experiments.

The plant cytokine, kinetin, a small molecule has been found to be a powerful agent that corrects *IKBKAP* mRNA splicing defects in FD cells by an unknown mechanism [26]. The effect of kinetin was shown to correct IKAP mis-splicing in several non-neuronal cells from FD patients and *in vivo* [23–28], but not yet in human-derived neurons. To this end, differentiated hESC-derived neurons were treated with 50 and 100 μM kinetin for 24 hours and RNA was extracted for RT-PCR analysis as shown in Fig 9A. Kinetin treatment as expected, corrected the FD splicing in FD neurons showing mostly the correct transcript isoform as compared to untreated control. Next we treated FD neurons with 100 μM kinetin for two days to investigate the kinetin effects on the expression of *IKBKAP* and on the potential IKAP associated/target genes by qRT-PCR analysis as shown in Fig 9B. In these experiments the total level of *IKBKAP* mRNA increased by more than 5 fold together with a shift of mis-spliced to correct-spliced mRNA, as shown by the higher WT transcript expression over the total *IKBKAP* (which includes the mRNA FD-splicing variant). Concomitantly, most of the candidate genes were also upregulated following kinetin treatment. These results suggest that the expression of the IKAP associated/target genes are indeed linked, probably depending on recovery of IKAP protein due to kinetin treatment in FD-neurons. Another possibility for the upregulation of IKAP related genes is that kinetin has a similar effect on the expression of these genes independent of IKAP itself, perhaps through co-TF regulation, as indicated in Fig 8B. To study the effect of kinetin treatment on IKAP protein expression and concomitantly on other IKAP functional related proteins such as Rab3a (target of RPH3A) and GRIA1, FD-neurons were treated with 100μM of kinetin for prolonged time periods of 4 weeks. The cells were fixed and prepared for immunofluorescence analysis under a confocal microscope using specific antibodies against these proteins. Representative confocal micrographs from these experiments are shown in Fig 9C–9M. While in untreated FD neuronal cultures the level of IKAP is very low (Fig 9C and 9D) at 4 weeks kinetin treatment IKAP fluorescence signal level is dramatically elevated under the same confocal scanning conditions as shown qualitatively and quantitatively in Fig 9E, 9F and 9G respectively. Together with this Rab3a and GRIA1 expression levels in FD neurons dramatically increased in response to kinetin treatment (Fig 9J and 9K) suggesting that potentially neuronal function associated with these proteins is restored by the treatment. Altogether, these results support the view that IKAP might be localized in vesicles which are probably involved in neuronal transport and synapse integrity in hESC-derived PNS neurons. These functions seem to be mostly affected in FD-hESC-derived PNS neurons probably reflecting some of the PNS dysfunction phenotypes observed in FD.

## Discussion

Here we describe a novel and unique *in vitro* experimental system that promotes the study of FD. We have characterized genotypically and phenotypically the PGD-HAD-4 (FD-hESC) cell line as a human FD model and demonstrated that regardless the FD mutation the pluripotency capacity and its potential to produce specifically neural crest like lineages and differentiated PNS like neurons in general seem not to be affected. Here we examined the levels of *IKBKAP* expression in FD hESC throughout neuronal PNS differentiation. It turns out that total *IKBKAP* levels in FD hESC are much lower than in WT hESC in all stages tested during the 12 weeks of neuronal differentiation. Moreover, *IKBKAP* levels in FD-hESC-derived mature neurons stage are the lowest. The work of Lee and coworkers found that in undifferentiated, pluripotent, WT and FD hiPSCs the levels of *IKBKAP* are not different and that following differentiation into neural crest the levels of WT *IKBKAP* isoform decline dramatically [5]. These findings differ from ours since in our model the PGD FD hESC at the pluripotent stage showed already a 6-fold difference in *IKBKAP* expression from the WT hESC control. The difference in *IKBKAP* splicing isoform levels at the pluripotent stage between the FD stem cell models hiPSC and PGD hESC may be due to the known existing genetic and epigenetic differences between hiPSCs and hESC [44, 45]. The grounds for these differences were attributed to the “epigenetic memory” resulting from the tissue origin of the founder cells of the hiPSC [46]. These studies indicate significant drawbacks of hiPSC compared to hESC, which may hamper their use for disease modeling. The first example of a clear difference in disease modeling between hiPSC and hESC was demonstrated by Urbach and colleagues, their data highlight a significant difference between FRAX-hESC and FRAX-iPSC with regard to their epigenetic state which influences the expression of the FMR1 gene [47]. This distinction might be a particular example of a general phenomenon of epigenetic differences between hESCs and hiPSCs. They conclude that the study of both hiPSC from patients and hESC carrying the same mutation should be the optimal approach to model human genetic disorders through cell culture. Our FD-hESC *in vitro* model enabled the study of the expression and function of IKAP in human FD-NCC and peripheral neurons contributing to our understanding of the complexity of the FD-phenotype. We showed that NCC-derived from the FD-hESC had a reduced cell migration capability as demonstrated by different migration assays. Several studies suggested that IKAP has a role in cell motility and migration; however the exact mechanism still awaits discovery. IKAP was described to associate with, and is needed for the membrane ruffle localization of filamin A, a protein capable of driving cell migration [48]. Alternatively, IKAP was suggested to interact with the MT monomer α-tubulin, one of the major dynamic structural component of the cytoskeleton, through its acetylation [49, 50] or through the regulation of the expression of the MTs destabilizing proteins as shown previously by us [51]. Our findings in this work suggest that impaired *IKBKAP* downregulation may be associated with an impaired NCC migration. In line with our observation, Studer and colleagues demonstrated a significant decrease in the migration of FD-hiPSC- versus control-hiPSC-derived NCC [23]. Analysis of differential gene expression between FD and WT during hESC neuronal differentiation revealed that the majority of changes in gene expression occurred at the final stages of the PNS neuronal differentiation. Remarkably, these differences in gene expression were not reflected by clear morphological differences between the FD and WT neuronal cultures. We assume that this discrepancy is related with the high density of neurons generated from hNP spheres that make it difficult to identify and isolate possible fine morphological features in FD neurons by image microscopy analysis. Nevertheless, we did detect significant differences in the FD-hESC-derived PNS neurons at the subcellular level using confocal microscopy showing a significant reduction in vesicular-like IKAP colocalization with known neuronal vesicular proteins indicating potential defects in key neuronal functions such as synaptic vesicular and neuronal transport in this model. Gene ontology analysis revealed that the main processes affected in FD are related to cytoskeleton organization, neurogenesis, axon guidance, synapse assembly, maintenance and electrophysiology properties. These affected cellular processes are the result of gene down regulation of GABA and glutamate receptor expression, calcium-dependent mechanisms, and intracellular trafficking. The upregulated gene expression differences were mainly related to extracellular-matrix signaling and organization. From these analyses we conclude that the majority of processes affected by *IKBKAP* downregulation in FD neurons are mostly related to the nervous system, and hence could not be detected in other studies based on non-neuronal cells. Previous transcriptome mRNA expression using differentiated *IKBKAP* downregulated neuroblastoma cell lines [52] found that late neuronal development, axonal projection, synapse formation and function were affected by IKAP depletion. In undifferentiated neuroblastoma, however, the main processes that were affected by *IKBKAP* downregulation included genes involved in cell signaling, cell adhesion, and neural crest migration [52]. Similar results based on neural crest-derived hiPSC from FD patients by Lee and co-workers revealed that among the 20 most decreased transcripts in FD neural crest precursors, were many genes involved in peripheral neurogenesis and neuronal differentiation [23]. Our platform of PNS neurons derived from FD-hESC cell line unlike the others, allows the study of function of IKAP in post mitotic differentiated neurons which is most relevant to the understanding of the basics behind the abnormal function of PNS neurons in FD. Comparing mRNA expression results from FD-hESC-derived PNS neurons with expression results obtain from FD 12-week-old human fetus brains, allowed us to establish the physiological relevance of our *in vitro* platform. It seems that our FD stem cell neuronal model derived from a PGD individual embryo shares the highest number of differentially expressed genes with the fetal FD brain sample in comparison with hiPSC and hOE-MSC FD models [23, 24]. In other words our model shows probably a greater similarity in the number of affected genes with the FD fetal brain sample than the other two FD stem cell-derived neural models. Moreover, this genome wide analysis between our in vitro FD-hESC PNS neurons and FD brain allowed us to further narrow the findings regarding important neuronal genes involved in the FD phenotype. The results revealed the genes that are mostly affected in the developing FD nervous system, like for example ion-gated channels specific genes for neuron pre and post-synaptic excitability. Approximately a 30% correlation was found between the genes in both *in vivo* and *in vitro* FD samples showing similar GO annotations for gene function and processes. To the best of our knowledge these results are the first to suggest a possible physiological context to the molecular changes found in FD-hESC PNS neurons and the developing FD brain supporting the future use of the FD-hESC PNS neurons as model to unravel the FD phenotype. Using this FD model we found that IKAP is present mostly in the cytosol of neurons, some in the nucleus and predominantly present in vesicular structures, much like typical synaptic vesicles. IKAP immunofluorescence staining in hESC-derived PNS neurons shows a similar expression pattern with the synaptic vesicle SV2 marker, which marks some of the neurotransmitter vesicles in neurons [53]. Moreover, IKAP was also partially colocalized with Rab3a, which is known to be associated with the priming of synaptic vesicles [54]. Strong support for these observations showing that IKAP is also a vesicular protein associated with neuronal transport in PNS neurons comes from our recent publication [21]. In this previous publication we attempted to elucidate the IKAP role during PNS development in the chick embryo and found that IKAP is required for proper axonal outgrowth, branching, and peripheral target innervation. Moreover, we demonstrate that IKAP colocalizes with activated JNK (pJNK), dynein, and **β**-tubulin at the axon terminals of dorsal root ganglia (DRG) neurons, and is probably involved in transport of specific target-signals required for transcription of JNK and NGF responsive genes in the nucleus [21]. From the above we can conclude that IKAP function in neurons may play an important role in neuronal vesicular transport and, for obvious reasons, this function is different from that played in fibroblasts probably throwing light on the mechanism behind the specificity of the FD phenotype. IKAP partial colocalization with Rab3a, implies that IKAP might be an element needed to facilitate/sort diverse synaptic vesicle transport subtypes or cargo. A previous study in yeasts supported IKAP's role in vesicular transport and exocytosis. It showed that the yeast IKAP homolog, Elp1p, has a cytosolic role in polarized exocytosis of post-Golgi secretory vesicles [55]. Elp1p was found to be physically associated with sec2p, a Rab3a human homolog, facilitating polarized exocytosis mediated by the yeast Rab protein [56]. The FD hESC-derived neurons showed constitutive IKAP deficiency throughout the differentiation period. To study the effects of *IKBKAP* downregulation on the potential target genes identified by our in vitro and in vivo double transcriptome analysis we performed knockdown of *IKBKAP* by siRNA in WT hESC PNS neurons. The immediate effect of siRNA downregulation of *IKBKAP* on KIF5A, GRIA1, SNCA and Rab3a may indicate that these genes may be more closely regulated by IKAP or linked to IKAP function than the rest of the genes tested. A possible connection between these downregulated genes has been suggested by Setou et al which showed that KIF5A binds GRIP1, an adaptor protein of GRIA1, which in turn, transports the AMPA receptor to its appropriate location along the neurite [43]. It was also recently shown that α-Synuclein (SNCA) membrane association is regulated directly by the Rab3a protein [57]. Surprisingly, DYNCI-1, SYP were upregulated following *IKBKAP* siRNA knockdown, emphasizing the difference between acute and prolonged deficiency of *IKBKAP* as in FD. To date, several compounds have been proposed as potential therapeutics for FD. Phosphatidylserine, was reported to modify *IKBKAP* splicing in mice, and Tocotrienols were reported to increase total *IKBKAP* transcript production in human FD cell lines [58, 59]. Kinetin has been shown to be one of the most potent drugs to correct *IKBKAP* alternative splicing and upregulate IKAP expression in FD cells [25, 26] although its therapeutic potential is uncertain due to possible side effects and toxicity in FD patients [60]. Investigation into the kinetin mechanism of action may be useful for further development of drug treatments for FD. Here we have shown that 1 day kinetin treatment of FD hESC-derived neurons elevated *IKBKAP* level by 6-7-fold and also elevated the entire related candidate gene target: KIF5A, RPH3A, GRIA1, DYNCI-1, SYP, CAMK2B, MAPT and SNCA by 2-8-fold. The exact way by which kinetin alters the splicing machinery is unknown, although previous studies indicate that the sequence of the splice donor site of exon 20 is required for activity. *IKBKAP* exon 20 ends with a CAA, rather than the consensus sequence CAG, and testing of kinetin on many exon 20 deletion constructs showed that the CAA sequence was required for kinetin activity probably reinforcing U1 snRNP-mediated 5’ss recognition site for correct exon 20 splicing of *IKBKAP* with the FD mutation [27]. Kinetin’s ability to modify aberrant splicing is not limited to *IKBKAP* [61]. Although kinetin helps to increase WT *IKBKAP* transcript level, the compound does not seem to influence the expression of a large proportion of genes as shown by genome wide gene expression analysis [42]. We have also shown that prolonged treatment with kinetin results in elevation in IKAP protein levels in FD neurons. It is still not clear from these experiments whether other proteins such as GRIA1 and Rab3a were elevated by direct influence of kinetin or via indirect pathways involving IKAP upregulation. The elevation of Rab3a and GRIA1 staining was observed in the cell neurites and for GRIA1 also in the cell nucleus probably indicating restoration of neuronal function in the FD neurons in response to kinetin treatment since Rab3a and GRIA1 have been shown to be associated with glutamate-AMPA receptors activity as mentioned above [43].

## Conclusions

In summary this work demonstrates that the NCC and PNS neurons derived from the unique PGD-FD-hESC line characterized in this study, may serve as a highly relevant cell model to study FD, reflecting in some way the phenotype of FD neurons *in vivo*. The use of this model allows to elucidate the molecular mechanisms involved in neuronal malfunction in FD and to identify potential drug targets and validate potential drugs for FD treatment that restore either IKAP or neuronal function in the FD neurons.

## Supporting Information

S1 Fig Characterization of FD-hESC. hESC derived from a FD-affected PGD embryo which were cultured in hESC culture conditions formed colonies with typical morphology (A; phase-contrast image) and were positive for alkaline phosphatase staining (B). Indirect immunofluorescence staining shows that the undifferentiated FD-hESC are immunoreactive with anti-Oct-4 (C; D, DAPI nuclei counter staining). Fluorescence immunostaining shows FD-hESC also expressing the pluripotent markers Tra-1-60 (E), Tra-1-81 (F), SSEA-3 (G), and SSEA-4 (H). A representative chromosome spread of the FD-hESC (46, XY) is presented (I), indicating a normal karyotype of the FD-hESC. Scale bars: A, B 200μm; C, D 20μm; E–H 100μm.
(TIF)

S2 Fig FD-hESC are pluripotent and can differentiate into progeny of the all embryonic germ layers. Differentiation *in vitro* was either spontaneous within embryoid bodies (EB) or controlled into neural precursor spheres. After 3 weeks of differentiation by either method, cells were plated for a further 2–7 days of differentiation. Immunofluorescence staining shows that the differentiated cells expressed β-tubulin III (A), muscle desmin (B), and Sox17 (C), representing differentiation into ectoderm, mesoderm, and endoderm, respectively. Teratoma tumors developed 6–14 weeks after inoculation of FD-hESCs under the testicular capsule of NOD-SCID mice. Hematoxylin-eosin stained histological sections of the tumors show neural rosettes (D), cartilage (E), and structures with columnar epithelium that includes goblet cells (F) representing differentiation into ectoderm, mesoderm, and endoderm, respectively. Scale bar: A-C 20μm; D-F 50μm.
(TIF)

S3 Fig Genetic characterization of *IKBKAP* in FD-hESC. F-PCR for the specific 2507+6T→C (2507M) FD allelic mutation was performed on the FD-hESC (FD) and HES1 (WT) hESC showing the specific mutation in the FD-hESC only (A). Further sequencing for the 3' splice site of exon 20 genomic area within the *IKBKAP* locus shows the T→C FD allelic mutation in FD-hESC (B). RT-PCR analysis for the expression levels of the mutant (Mut; abnormally spliced) and wild type (WT) *IKBKAP* transcripts was performed on various stages of pluripotent and differentiated FD and WT cells, as indicated (C). Mutant *IKBKAP* transcripts were not observed in any of the samples of the WT hESC. Levels of the mutant *IKBKAP* transcript were higher in FD-hESC-derived NPs in comparison to the levels in spontaneously multi lineage differentiated EB.
(TIF)

S4 Fig Differentiation of FD-hESC-derived NP clusters into NCC. Schematic presentation of the steps for the derivation of NCC from hESC-derived NP clusters is shown in (A). hESC-derived NP clusters are platted and cultured on fibronectin in the presence of bFGF and EGF. After 24h, extensive migration of cells is observed. The migrating cells are selectively collected and cultured for further propagation. Migratory cells represent a uniform cell population with a protein and gene expression profile and differentiation characteristics of NCC. Migratory cells are positively immunostained for the typical NCC markers HNK1 (B, E), Ap2α (C, D and F) and Sox9 (G). FACS analysis shows that over 75% of the migratory cells express p75, a typical NCC marker and 64% of cells are positive for HNK1 (H). Gene expression analysis by qRT-PCR of the migratory cells show elevated expression of key NCC transcription factors such as *Pax3*, *Snail* and *Slug* along with low expression of the transcription factors *Sox2* and *Pax6* which are typically expressed by multipotent early hNP (I).
(TIF)

S5 Fig Differentiation of FD hESC into PNS neurons. Upper panel shows WT and FD-hESC differentiation process following the PNS neuronal differentiation protocol for a period of 8 weeks (A-J). (K-L) show 10 weeks fully differentiated WT and FD-hESC PNS neurons respectively. (A-B) show respectively WT and FD pluripotent hESC colonies grown on supporting human foreskin feeder cells. (C-D) show respectively bright filed images of WT and FD-hESC derived NPs sphere following 1 day of attachment on Laminin/ Poly-D-Lysine coated glass cover slips. (E-F) high magnification micrographs of E and F, showing outgrowing and migrating neurons for WT and FD respectively. (G-H) show respectively WT and FD cultures expressing the post mitotic neuronal marker β3-tubulin in 24 hours old neurites outgrowing from 8 weeks old WT and FD NPs. (I-J) show outgrowing neurons expressing the neuronal PNS marker Peripherin in WT and FD. (K-L) show 10 weeks mature neuronal cultures expressing β3-tubulin in WT and FD exhibiting similar classic PNS neuronal morphologies. Scale bars are indicated in representative images.
(TIF)

S1 Supporting Information Extended experimental procedures and methods for supplementary figures. (DOCX)

S1 Table GO analysis groups of differential gene expression between WT and FD-hESC derived PNS neurons. The major enriched GO analysis groups from the cDNA array chip showing processes (A) and gene functions (B). The enrichment groups are arranged by number of downregulated and upregulated genes detected in each group. Only genes above 2-fold change are included. FDR q-value (adjusted p-values of result significance), Enrichment = ((number intersection genes / input genes) / (total number of genes of specific GO term / background genes)).
(XLSX)

S2 Table List of all of the affected genes described in GO analysis of differential gene expression between WT and FD-hESC derived PNS neurons (see Table). (XLSX)

S3 Table List of the common genes from the wide genome transcriptome analysis that are differentially expressed in FD-hESC-derived PNS neurons, FD fibroblasts derived iPSC, FD-hOE-MSC and in FD Fetal brain as represented in Fig 6C. Cross-referencing genes with their expression levels difference of >2 fold change for hESC p<0.05 or >1.5 fold change p<0.05 for iPSC and hOE-MSC between WT and FD were considered for analysis.
(XLSX)

S4 Table List of all of the affected genes described in GO analysis of differential gene expression between hESC-derived neurons and embryo brains of WT and FD origin (see Table 1). (XLSX)

S5 Table Ion gated channel genes altered expression in FD hESC-derived neurons. The table shows the fold change of differential expression of specific genes within the group for calcium, potassium, and sodium gated channel genes between wt and FD hESC-derived PNS neurons. Only genes above 2-fold change are included in the list.
(XLSX)
