# Supplementary figures and images for: Familial Dysautonomia (FD) Human Embryonic Stem Cell Derived PNS Neurons Reveal that Synaptic Vesicular and Neuronal Transport Genes Are Directly or Indirectly Affected by IKBKAP Downregulation
Source: PLoS One. 2015 Oct 5;10(10):e0138807. doi: 10.1371/journal.pone.0138807 (PMC4593545; doi:10.1371/journal.pone.0138807)

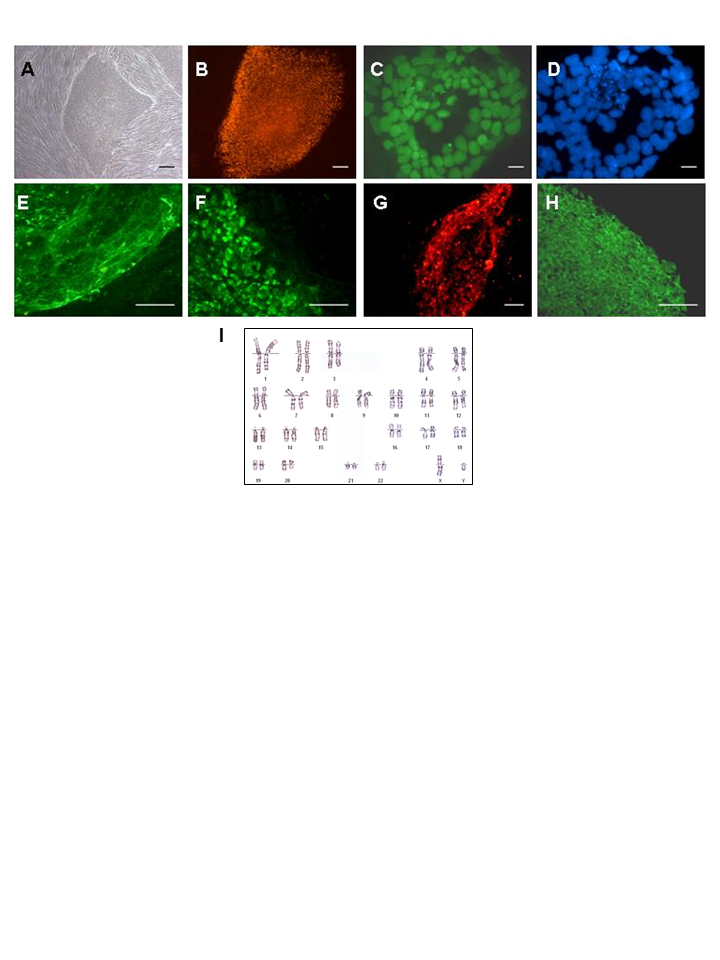

Supplement: S1 Fig — hESC derived from a FD-affected PGD embryo which were cultured in hESC culture conditions formed colonies with typical morphology (A; phase-contrast image) and were positive for alkaline phosphatase staining (B). Indirect immunofluorescence staining shows that the undifferentiated FD-hESC are immunoreactive with anti-Oct-4 (C; D, DAPI nuclei counter staining). Fluorescence immunostaining shows FD-hESC also expressing the pluripotent markers Tra-1-60 (E), Tra-1-81 (F), SSEA-3 (G), and SSEA-4 (H). A representative chromosome spread of the FD-hESC (46, XY) is presented (I), indicating a normal karyotype of the FD-hESC. Scale bars: A, B 200μm; C, D 20μm; E–H 100μm. (TIF) [file pone.0138807.s001.tif]

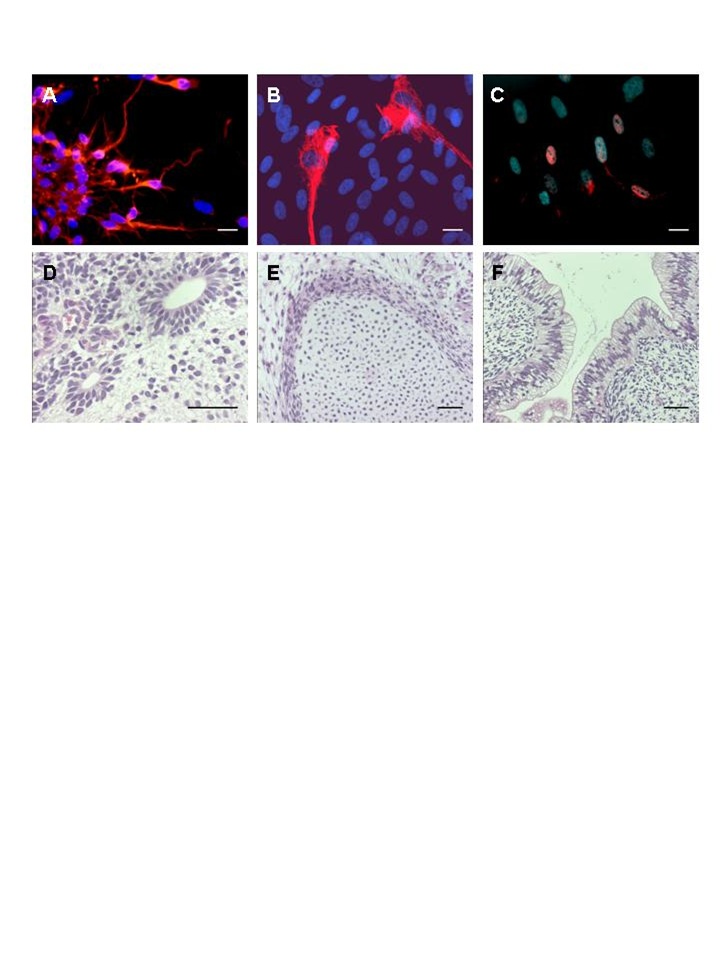

Supplement: S2 Fig — Differentiation in vitro was either spontaneous within embryoid bodies (EB) or controlled into neural precursor spheres. After 3 weeks of differentiation by either method, cells were plated for a further 2–7 days of differentiation. Immunofluorescence staining shows that the differentiated cells expressed β-tubulin III (A), muscle desmin (B), and Sox17 (C), representing differentiation into ectoderm, mesoderm, and endoderm, respectively. Teratoma tumors developed 6–14 weeks after inoculation of FD-hESCs under the testicular capsule of NOD-SCID mice. Hematoxylin-eosin stained histological sections of the tumors show neural rosettes (D), cartilage (E), and structures with columnar epithelium that includes goblet cells (F) representing differentiation into ectoderm, mesoderm, and endoderm, respectively. Scale bar: A-C 20μm; D-F 50μm. (TIF) [file pone.0138807.s002.tif]

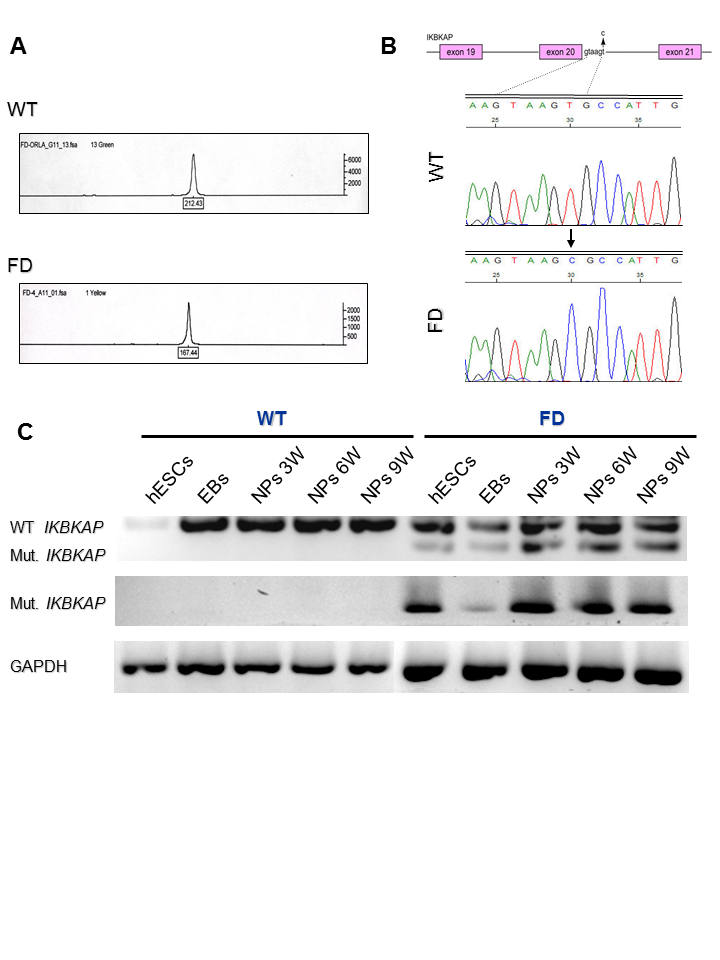

Supplement: S3 Fig — F-PCR for the specific 2507+6T→C (2507M) FD allelic mutation was performed on the FD-hESC (FD) and HES1 (WT) hESC showing the specific mutation in the FD-hESC only (A). Further sequencing for the 3' splice site of exon 20 genomic area within the IKBKAP locus shows the T→C FD allelic mutation in FD-hESC (B). RT-PCR analysis for the expression levels of the mutant (Mut; abnormally spliced) and wild type (WT) IKBKAP transcripts was performed on various stages of pluripotent and differentiated FD and WT cells, as indicated (C). Mutant IKBKAP transcripts were not observed in any of the samples of the WT hESC. Levels of the mutant IKBKAP transcript were higher in FD-hESC-derived NPs in comparison to the levels in spontaneously multi lineage differentiated EB. (TIF) [file pone.0138807.s003.tif]

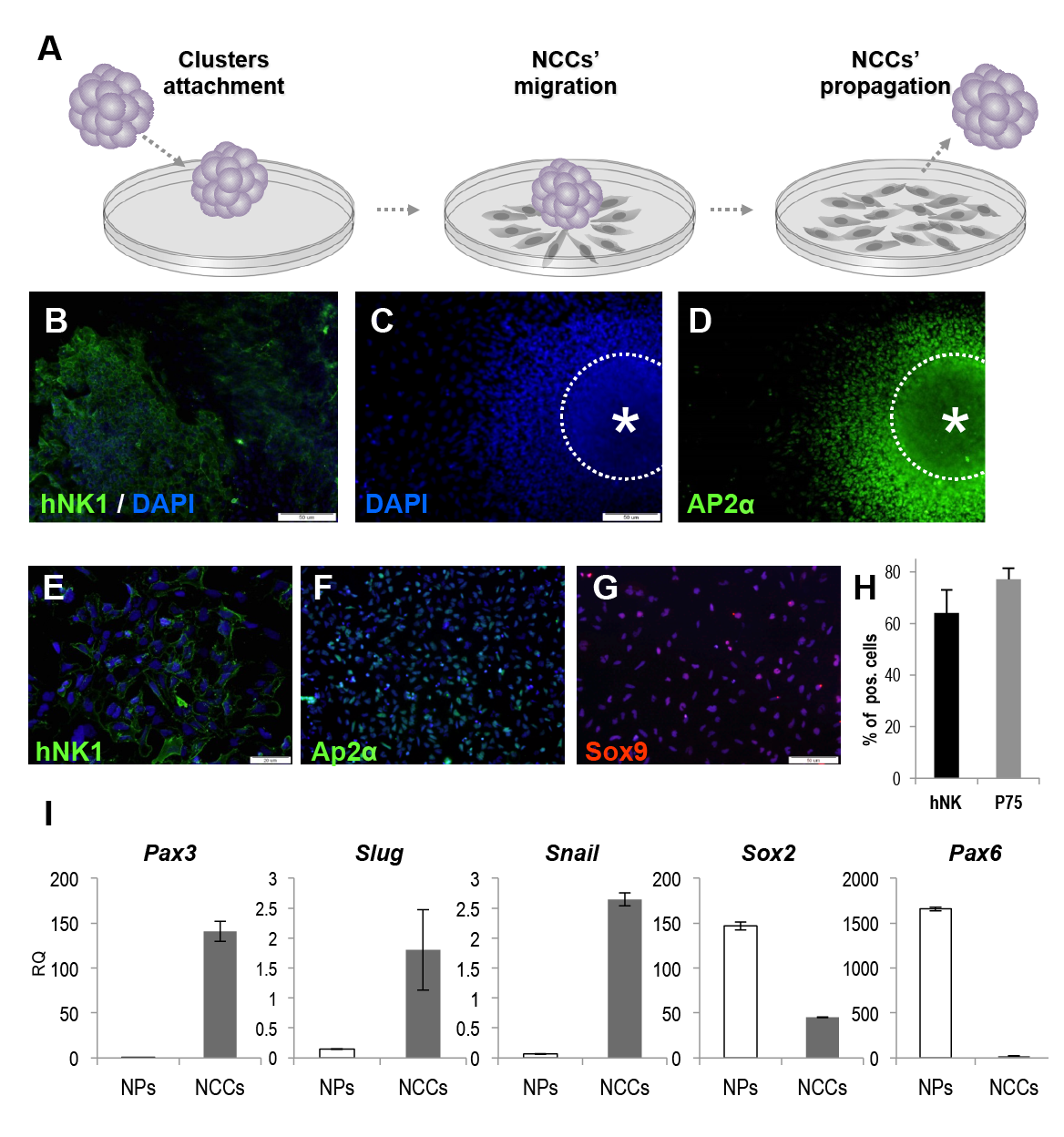

Supplement: S4 Fig — Schematic presentation of the steps for the derivation of NCC from hESC-derived NP clusters is shown in (A). hESC-derived NP clusters are platted and cultured on fibronectin in the presence of bFGF and EGF. After 24h, extensive migration of cells is observed. The migrating cells are selectively collected and cultured for further propagation. Migratory cells represent a uniform cell population with a protein and gene expression profile and differentiation characteristics of NCC. Migratory cells are positively immunostained for the typical NCC markers HNK1 (B, E), Ap2α (C, D and F) and Sox9 (G). FACS analysis shows that over 75% of the migratory cells express p75, a typical NCC marker and 64% of cells are positive for HNK1 (H). Gene expression analysis by qRT-PCR of the migratory cells show elevated expression of key NCC transcription factors such as Pax3, Snail and Slug along with low expression of the transcription factors Sox2 and Pax6 which are typically expressed by multipotent early hNP (I). (TIF) [file pone.0138807.s004.tif]

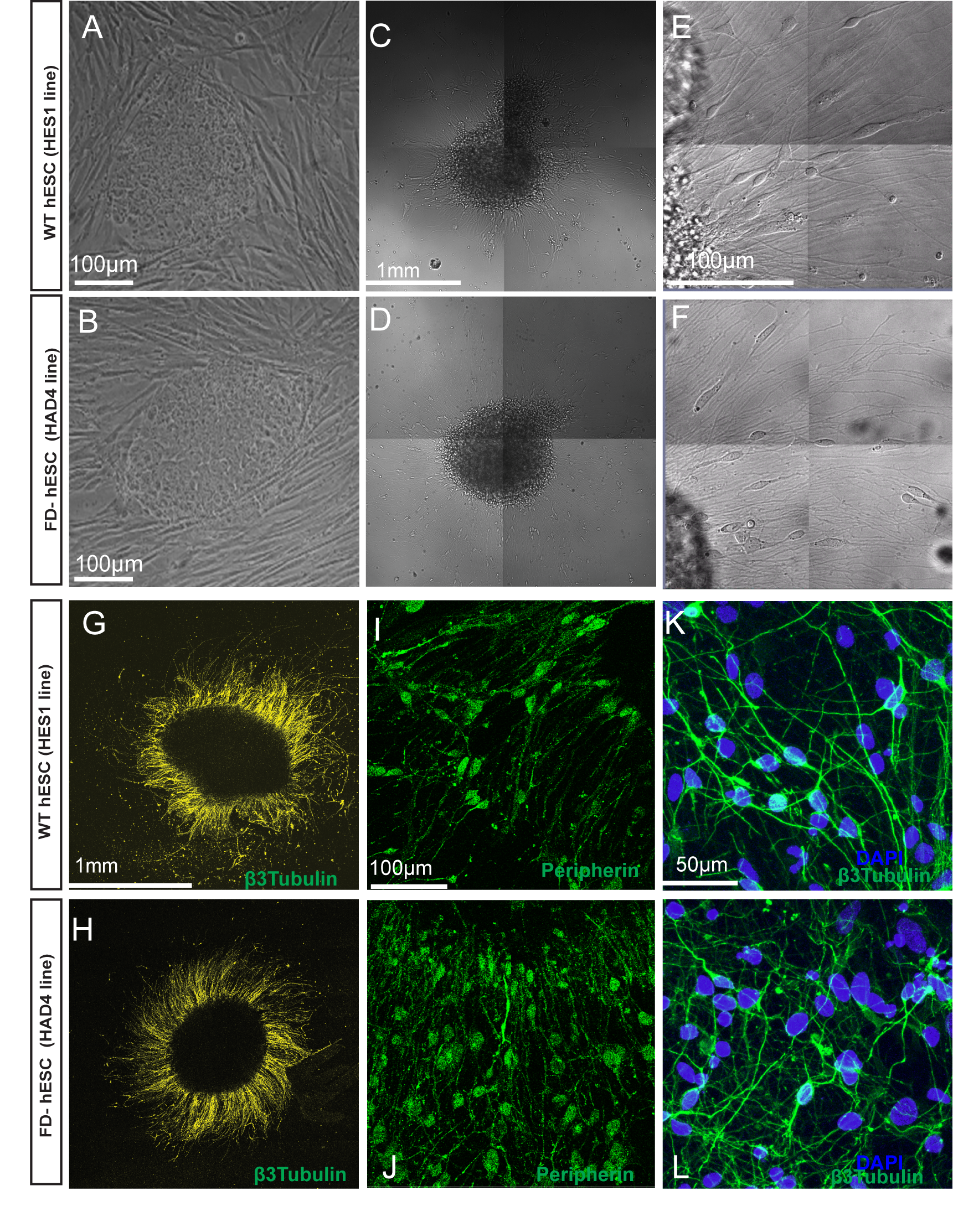

Supplement: S5 Fig — Upper panel shows WT and FD-hESC differentiation process following the PNS neuronal differentiation protocol for a period of 8 weeks (A-J). (K-L) show 10 weeks fully differentiated WT and FD-hESC PNS neurons respectively. (A-B) show respectively WT and FD pluripotent hESC colonies grown on supporting human foreskin feeder cells. (C-D) show respectively bright filed images of WT and FD-hESC derived NPs sphere following 1 day of attachment on Laminin/ Poly-D-Lysine coated glass cover slips. (E-F) high magnification micrographs of E and F, showing outgrowing and migrating neurons for WT and FD respectively. (G-H) show respectively WT and FD cultures expressing the post mitotic neuronal marker β3-tubulin in 24 hours old neurites outgrowing from 8 weeks old WT and FD NPs. (I-J) show outgrowing neurons expressing the neuronal PNS marker Peripherin in WT and FD. (K-L) show 10 weeks mature neuronal cultures expressing β3-tubulin in WT and FD exhibiting similar classic PNS neuronal morphologies. Scale bars are indicated in representative images. (TIF) [file pone.0138807.s005.tif]
