# Supplementary material for: Familial Dysautonomia (FD) Human Embryonic Stem Cell Derived PNS Neurons Reveal that Synaptic Vesicular and Neuronal Transport Genes Are Directly or Indirectly Affected by IKBKAP Downregulation
Source: PLoS One. 2015 Oct 5;10(10):e0138807. doi: 10.1371/journal.pone.0138807 (PMC4593545; doi:10.1371/journal.pone.0138807)
Supplement: S1 Supporting Information — (DOCX) [file pone.0138807.s006.docx]

**S1 Supporting Information**

**Characterization of Pluripotency and FD mutation genotype of the FD-hESC line**

A human *in vitro* fertilized embryo that was diagnosed to be affected by FD was recruited for this study from our PGD program. A hESC line was established from the laser-dissected ICM of the embryo, as previously described ([17](#_ENREF_17)). The colonies of the FD-hESC line displayed the typical characteristics of hESC colonies (Fig. S1A). The cells of the FD-hESC line showed alkaline phosphatase activity (Fig. S1B). Immunophenotyping of intact colonies demonstrated that the cells within them expressed the typical markers of pluripotent stem cells such as Oct4, Tra-1-60, Tra-1-81, SSEA3 and SSEA4 (Fig. S1C-H). The cell line showed a normal diploid karyotype (Fig. S1I). The pluripotent potential of the FD-hESC line was demonstrated by its capability to differentiate into progeny representing the three embryonic germ layers both *in vitro* and *in vivo* (Fig. S2).

The FD-hESC line was derived from an embryo that was donated by a couple carrying the 2507+6T→C FD mutation. Allele specific and linkage analysis by Fluorescence PCR showed that the two blastomeres that were removed from the embryo during PGD, as well as the FD-hESCs that were derived, were homozygous for the 2507M mutation (Fig S3A). Furthermore, sequencing of the genomic area of the mutation, demonstrated that the *IKBKAP* gene from the FD-hESCs had the specific transition from T-to-C at position 6 of the 5’ splice site on intron 20 (Fig. S3B). The expression levels of the wild type (WT) and mutant transcripts of *IKBKAP* mRNA were tested in undifferentiated hESCs, in embryoid bodies (EBs) representing spontaneous multi lineage differentiation, and in neural precursors (NPs) derived from WT (HES1 cell line) and from the FD-affected hESCs (Fig S3C). *IKBKAP* mutant transcripts were found only in the FD-hESCs derived samples. Moreover, we showed that the ratio of expression of mutant and WT transcripts of *IKBKAP* differed in various hESC-derived differentiated cell populations. The mutant transcript was more highly expressed in the FD hESC-derived NPs compared to undifferentiated FD hESCs or differentiating EBs. This finding is in line with the preferential expression of the mutant *IKBKAP* mRNA in the brain and nervous system of FD patients and suggests that the FD hESCs may serve as a reliable model of the disease.

**Material and Methods**

**Human embryos and PGD procedure**

PGD embryos were donated by a couple undergoing IVF treatment at the IVF unit of the Hadassah Medical Center of The Hebrew University. Embryos that were not diagnosed as genetically normal were recruited for this study subject to informed consent of the couples. The study was approved by the ethical committee at the Hadassah Medical Center as well as the Israeli Ministry of Health National Helsinki Committee for Genetic Research in Humans.

PGD procedure was done as previously described (Turetsky et al., 2008) at the IVF unit at the Hadassah Medical Center of The Hebrew University. Blastomere biopsy was performed on embryos that contained at least four blastomeres. One or two cells were removed from the embryos for PCR analysis. The cells were washed five times in PBS, supplemented with 4% serum substitute supplement (SSS; both from Irvine Scientific), transferred to microcentrifuge tubes (one cell per tube) and were delivered on dry ice to the lab of the Department of Human Genetics in the Hadassah Medical Center where they were individually diagnosed.

**Genetic diagnosis of single blastomeres**

DNA was extracted and multiplex fluorescent PCR (F-PCR) was used to analyse the WT allele of *IKBKAP* (2507N), the allele-specific mutation of FD (2507M), and to three FD linked polymorphic markers (D9S1828, D9S309 and D9S1801) (Harper et al., 2002) using ABI prism and the Genotyper softweares (Applied Biosystems).

**ICM isolation and derivation of hESCs**

Embryo culture and ICM isolation was preformed as previously described (Turetsky et al., 2008). The isolated ICM was plated on Mitomycin-C inactivated human foreskin and cultured in KO medium supplemented with 4 ng/ml bFGF.

After 5–8 days, small tightly-packed cells were dissected mechanically from the ICM or whole embryo outgrowths and plated on fresh feeders. Colonies of cells resembling hESCs gradually appeared within a few passages. Routine passaging of putative hESCs was performed by mechanical dissociation of the colonies into small clusters and plating on fresh feeders. Clusters of early passage hESCs were cryopreserved by vitrification according to our published protocol or by conventional slow cooling in 90% FCS (Biological Industries, Kibbutz Beit Haemek, Israel) and 10% DMSO (Sigma). For the development of bulk cultures and a large number of cells, the hESCs were passaged by enzymatic treatment. Bulk passaging was performed up to 12 weeks.

**Confirmation of the diagnoses in the hESC lines**

F-PCR analysis of the specific mutation and the polymorphic markers as above was used to confirm the diagnosis of FD in the FD-hESCs.

We also performed a direct sequencing of the 5'SS of exon 20 of *IKBKAP* gene. To avoid fibroblast cells contamination FD-hESCs and WT-hESCS derived EBs were cultured for long period (4 weeks) in EBs medium. Cells were then collected and genomic DNA was extracted from them by the Wizard Genomic DNA Purification Kit (Promega, Madison, WI) according to the manufacturer’s protocol. DNA was dissolved in ddH2O and was kept for O/N at 4ºC. PCR amplification reaction for the exon 19 and intron 20 boundaries on *IKBKAP* gene was performed on 100ng extracted DNA using specific primers and with Taq DNA polymerase. Amplification conditions were as follows: denaturation at 95ºC for 30 seconds, annealing at 56ºC for 30 seconds, and extension at 72ºC for 45 seconds, for 30 cycles. Products were analyzed on a 1.5% agarose gel and visualized by ethidium bromide staining. A single band product of 324 bp was observed in the agarose gel. PCR amplification products were purified using the QIAquick PCR Purification Kit (Qiagene, Hilden, Germany). 10µl of PCR fragments (50ng/µl) were sent for sequencing at the Center for Genomic Technologies, The Alexander Silberman Institute of Life Science, The Hebrew University, using 2pmoles/µl (2µM) of each primer.  Sequencing was done using the ABI Prism 3730xl DNA Analyzer and the ABI’s Data collection and Sequence Analysis softwares (Applied Biosystems).

**Characterization of hESCs**

The undifferentiated state of hESCs and hiPSCs can be demonstrated by immunofluorescence staining or FACS analysis for the expressiom of Oct-4, SSEA-3 SSEA-4 , Tra-1-60 and Tra-1-81.

Vector Red Alkaline Phosphatase substrate kit I (Vector Laboratories Inc.) was used for the detection of alkaline phosphatase activity within intact colonies on feeders according to the manufacturer's instructions.

For karyotype analysis, hESC/hiPSC colonies that were expanded with Collagenase type IV were incubated for 2–4 h with Demecolcine (0.2–0.3µg/ml) or Colcimide (1µg/ml). The hESC colonies were then removed from the feeders, dissociated with trypsin (Biological Industries), centrifuged at 170×g for 5min, resuspended in a hypotonic solution and incubated for 10 min in 37°C, followed by fixation with 3:1 methanol/acetic acid. The karyotype of 20 metaphases was analysed using the G-banding method.

**Analysis of pluripotency by in-vitro differentiation**

Colonies of undifferentiated cells were removed from the feeder with 1 mg/ml Collagenase type IV and cultured in suspension as embryoid bodies (EBs) or NPs. For the development of EBs, the hESC free-floating clusters were cultured 3 weeks in EBs medium. After the 3 weeks of suspension culture, the EBs were characterize  for the expression of endodermal (Sox17) and mesodermal (Desmin) markers and the NPs were characterized  for the expression of ectodermal markers (β-tubulin III) by immunofluorescence staining.

**Teratoma formation** **assay**

Pluripotency of hESCs was assessed by teratoma formation in nonobese diabetic severe combined immunodeficient (NOD SCID) mice (Harlan, Jerusalem, Israel) following the approval of the Institutional Ethical Committee for Care and Use of Animals. Clusters of ~200 hESCs were mechanically removed from the feeders and injected under the testes capsule of 6-week-old NOD SCID mice (10–15 clumps per testis). After 6–14 weeks, the resulting tumors were removed, fixed in neutral buffered 4% formalin, embedded in paraffin and examined histologically after hematoxylin and eosin staining.

**Migration assays:**

**Wound healing assay**

Wound healing experiments were performed on confluent cultures of NCCs (P1 or P2) cultured for 4-5 days in CDM with bFGF and EGF (20ng/ml). Following confluency, a scratch was mechanically made to produce a gap within the cell layer. The medium was replenished and was supplemented with fibronectin (10μg/ml) to allow cell migration into the gap. The cells were photographed at the gap area to visualize and measure changes in the gap width due to cell migration, at the beginning and 3, 5, or 7 hours after the wound was made. Areas were measured using the ImageJ software (NIH; <http://imagej.nih.gov/ij/index.html>). The recovery index of the gaps was determined by the ratio between the gap area, measured at several points along the gap, in the images from the initial and final time points.

**Time-lapse assay**

For time-lapse studies, 10,000-30,000 NCCs were plated at low density on 35mm glass-bottom dishes (20mm; MatTek, Ashland, MA) precoated with poly-d-lysine /fibronectin. Cells were cultured in CDM with bFGF and EGF over night. Medium was replenished and cells were allowed to migrate at 37^o^C at 5% CO_2_, 5% O_2_, and 90% nitrogen in a humid incubation chamber (Solent Scientific, Segensworth, UK) for 8 or 15 hours. Cell movements were monitored using FV1000 Fluoview confocal microscope (Olympus, Melville, NY) and images were acquired at low magnification (10x/0,25) objective with a sampling interval of 5 minutes. The exposure time was kept constant for all positions and all time points. Images were processed with the FV1000 software (Olympus, Melville, NY), exported in TIFF format and further analyzed using the MetaMorph software (Molecular Device Inc., Sunnyvale, CA). The movement of 10-20 individual cells were analysed in each assay. Motility and persistence of migratory directionality were determined by tracking the positions of cell nuclei using the Track Point function of MetaMorph. Motility was calculated as velocity (μm/min). The directionality index was calculated by D/T ratios represent the ratio of the direct distance from start to end point [D] divided by the total track distance [T].

**RNA extraction and cDNA production**

Total RNA was extracted with Trisol reagent (Invitrogen) or with RNeasy Mini Kit (Qiagene). Trizol or Buffer RLT (Trisol or RNeasy respectively) were applied directly into the culture dish to avoid loss of RNA. Samples that were previously treated with siRNA were isolated using RNeasy to avoid contamination of the siRNA products. In some cases, in order to eliminate a possible contamination with genomic DNA, 0.1 U/ml DNAse I (Ambion) was applied according to the manufacturer’s protocol. RNA was isolated according to manufactures’ manuals and eluted in 30μl RNAse-free pure water. The concentration of total RNA was measured using a Nano Drop Spectrophotometer (Nano Drop Technologies, USA). Total RNA (300 ng) was reverse-transcribed into complementary DNA (cDNA) with Reverse It 1^st^ Strand kit (ABgene) using oligo-dT as a primer according to the manufacturer's instructions.

**PCR reaction**

PCR reaction was carried out using ReddyMix Master Mix (ABgene) according to manufacture instruction. PCR products were analyzed by electrophoresis on 1% agarose gel.

**Table of qRT-PCR primers:**

| **Gene (Protein)** | | **Sequence 5’−>3’** | **Product size (bp)** |
| --- | --- | --- | --- |
| IKBKAP | Forward | GTTCATCATCGAGCCCTGGTTTTAG | 324 |
|  | Reverse | GCCACCTAAAACCCACATGTGT |  |

**Quantitative real-time PCR**

TaqMan (Life technologies) assay was performed in StepOne™ 96well Real-Time PCR System using TaqMan® Gene Expression Master Mix (2✕) in a MicroAmp® Fast Optical 96-Well Reaction Plate with Barcode. Data was analyzed using step one program and data assist (Life technologies). All gene, TaqMan probes were Fam™ labeled except IKAP-Total, which was labeled with Vic™, HPRT gene was used as endogenous gene control. All quantitative real-time PCR TaqMan analysis are presented as representative results in triplicates of 2-3 biological repeats.

**The following TaqMan probes were used:**

| **Assay ID** | **Gene Symbol** | **Gene Name** | **Species** | **Amplicon Length** |
| --- | --- | --- | --- | --- |
| Hs00365799_m1 | CAMK2B | calcium/calmodulin-dependent protein kinase II beta | Human | 62 |
| Hs00902194_m1 | MAPT | microtubule-associated protein tau | Human | 59 |
| Hs00863129_m1 | MARCH4 | membrane-associated ring finger (C3HC4) 4 | Human | 160 |
| Hs00189392_m1 | DYNC1I1 | dynein, cytoplasmic 1, intermediate chain 1 | Human | 68 |
| Hs00934263_m1 | RPH3A | rabphilin 3A homolog (mouse) | Human | 58 |
| Hs00192120_m1 | KIF5A | kinesin family member 5A | Human | 70 |
| Hs00165309_m1 | MYO5A | myosin VA (heavy chain 12, myoxin) | Human | 81 |
| Hs00181348_m1 | GRIA1 | glutamate receptor, ionotropic, AMPA 1 | Human | 86 |
| Hs01103383_m1 | SNCA | synuclein, alpha (non A4 component of amyloid precursor) | Human | 62 |
| Hs00300531_m1 | SYP | synaptophysin | Human | 63 |
| Hs01552822_m1 | NTNG1 | netrin G1 | Human | 61 |
| Hs00932050_m1 | IKBKAP (WT, Fam™,) |  | Human | 105 |
| Hs00932026_m1 | IKBKAP(total-red Vic™,) |  | Human | 86 |
| Hs99999909_m1 | HPRT1 | hypoxanthine phosphoribosyltransferase 1 | Human | 100 |

When TaqMan probes were not used quantitative real-time PCR was carried out using Absolute Blue QPCR SYBR® Green ROX Mix (Thermo Scientific, ABgene, UK) with 2µM each of the sense and antisense primers. Real-time PCR was performed under linear conditions using the following thermal cycle profile: Enzyme activation 950C 15min, followed by 50 cycles of: Denaturation 95°C 15 seconds, Annealing 56°C 30 seconds, Extension 72°C 30 seconds, followed by melt curve program: 72°C to 95°C linear ascend. Amplification was monitored and analyzed by measuring fluorescent dye SYBR Green I, which fluoresces after binding of the double stranded DNA. The overall fluorescence increases proportionally to the double stranded DNA concentration. 4 fold dilutions of total cDNA were used for external standards. After amplification, melting curves of the PCR products were acquired to demonstrate product specificity. Gene of Interest (GOI) cDNA levels are expressed relative to the ribosomal gene RS9 cDNA levels. All quantitative real-time PCR analysis are presented as representative results in triplicates of 2-3 biological repeats,

**Table of qRT-PCR primers:**

| **Gene (Protein)** | | **Sequence 5’−>3’** | **Product size (bp)** |
| --- | --- | --- | --- |
| IKBKAP TOTAL (correct+ mis-spliced) | Forward  (Exon 18) | GTTGACAACCCATTCCCATA | 118 |
|  | Reverse  (Exon 19) | CACTTTCCGCAGAACTTCCC |  |
| IKBKAP WT (Correct) | Forward (Exon 19) | TTCACGGATTGTCACTGTTG | 122 |
|  | Reverse  (Exon 20) | TGTCCAACCACTTCCGAATC |  |
| IKBKAP FD  (mis-spliced) | Forward (BRIDGE of exons 19-20) | CACAAAGCTTGTATTACAGA | 172 |
|  | Reverse  (Exon 21) | CTTAGGGTTATGATCATAAA |  |
| RS9 | Forward | GCCCATACTCGCCGATCA | 61 |
|  | Reverse | CGGAGACCCTTCGAGAAATC |  |

**Protein preparations and western blot analysis**

Proteins were extracted from cell pellets using Ripa buffer (Sigma-Aldrich Corp., Israel). Protein concentrations were checked using BCA kit (Pierce Biotechnology, IL, USA). For Western analysis, 30 mg proteins were loaded on 10% acrylamide gels or 4– 15% gradient gels (Bio Rad) in Tris-glycine buffer. Proteins were transferred to nitrocellulose membrane and blocked in 3% BSA (bovine serum albumin) in TBST for 1 hr. Primary antibodies used: Mouse monoclonal anti hIKAP (BD Biosciences, Franklin Lakes, New Jersey,USA 1:500), rabbit polyclonal anti hIKAP (Santa Cruz 1:500), mouse anti βactin ((Sigma-Aldrich Corp., Israel 1:1000), mouse anti βtubulin ((Sigma-Aldrich Corp., Israel 1:1000) were applied for 1 hr at room temperature. Secondary antibody was Donkey anti-mouse and Donkey anti-rabbit all HRP conjugated (at 1:10000) (Jackson Immuno Research laboratories, West Grove, PA, USA). For ECL, Super signal kit (Pierce Biotechnology, IL, USA) was used.

**cDNA microarrays**

Total RNA was extracted with Trisol reagent (Invitrogen), according to reagent’s protocol, treated and hybridized to DNA microarrays (Affymetrix GeneChipH Human Gene 1.0 ST arrays) according to the instructions manual, as described in the Affymetrix website (http://www.affymetrix.com). We used a total of 10 chips, two biological duplicates of FD and wt early neurons and three biological triplicates of FD and WT mature neurons.

**cDNA microarrays data analysis**

Data obtained from microarray experiments was analyzed using Microarray analysis was performed on CEL files using PartekH Genomics Suite TM, version 6.5 Copyright _ 2010 (http://www.partek. com). Data were normalized and summarized with the robust multi-average method (3), followed by analysis of variance (ANOVA). Cluster analysis of the array was obtained by PartekH Genomics Suite TM. Gene expression data were sorted using cutoffs of p<0.05 under FDR (false discovery rate). Data was also analyzed using Expander 6 (Lab Shamir R, Tel-Aviv University PMID 16176576) with a cutoff of 2 fold expression between wt and FD samples. Gorilla (4), was used to identify and visualize enriched GO terms in ranked gene lists. String (5), was used to identify protein interaction and known networks. Potential Transcription factors targets on selected genes were analgised using Prima (Promoter Integration in Microarray Analysis), (6) module in Expander 6 (7).

**Statistical analysis**

The significance of differences between samples control and treated was calculated using the two-tailed t-test. A p value of < 0.05 was used as the minimum criteria for statistical significance.

**Human fetuses and tissue sample collection**

Two human male 12 weeks fetuses were obtained from abortion procedures performed after approval of the hospital abortion committee. The study was approved by the ethical IRB of Hadassah Hebrew University Medical Center and the Israel Ministry of Health. The parents signed the inform consent for donation of tissues from the aborted fetuses for research. The pregnancy of the FD embryo was terminated since it was found to be homozygote for the FD mutation. The dissected tissues were immediately snap frozen in liquid nitrogen and then stored at -70°C until use.

**Brain RNA extraction**

Total mRNA was extracted from whole brain of normal and FD embryos, using Tri-reagent (Sigma) according to the manufacturer's protocol. Approximately 1 µg RNA of each sample was reverse transcribed using the M-MLV Reverse Transcriptase (Promega) and random primers in a 20 µl reaction mixture.

RNA integrity was assessed using an Agilent 2100 Bioanalyser (Palo Alto, CA). Samples with an RNA integrity number (RIN)> 7 were used for the analysis.

**RNA labeling, Hybridization and Agilent cDNA Microarray Scanning**

200 ng of total RNA for each fetal brain sample was amplified and labeled with a fluorescent dyes (Cy3 or Cy5) using the Low RNA Input Linear Amplification & Labeling kit (Agilent). The quality of the resulting labeled cRNA was measured using a Nanodrop ND-100 spectrophometer. The Cy3 and Cy5 labeled cRNA were hybridized to the Agilent Whole Human Genome Oligo Microarray V2 (design 026652, Agilent Technologies, USA) for 17 hours at 65°c in an Agilent DNA-Microarray Hybridization Oven. The arrays were later washed using the Gene Expression Wash Buffer Kit. The Microarray was scanned using the Agilent High-Resolution Microarray Scanner and the data was extracted from the resulting images using the Feature Extraction 10.7 software.

**Array Data Analysis**

Data analysis was carried out using LIMMA(8) software package, available from CRAN site (<http://www.r-project.org>). The Cy5 and Cy3 intensities within each array were normalized using the smoothing (LOESS) function, while no background correction was applied. To identify differentially expressed genes, a parametric empirical Bayesian approach implemented in LIMMA was used (9). A moderated t test was performed in parallel, with the use of a false discovery rate (10) correction for multiple testing. LIMMA calculated an emission intensity A value [A = (log2(Cy5) × Cy3)/2] where Cy5 and Cy3 are the normalized emission intensities of each feature. P value < 0.05 confidence level has been used to pinpoint those significantly differentiated genes. Genes had to have an A-value (average expression level for the gene across all arrays and channels) of more than 8.5, thus avoiding faint emissions.

**References**

1 Turetsky, T., Aizenman, E., Gil, Y., Weinberg, N., Shufaro, Y., Revel, A., Laufer, N., Simon, A., Abeliovich, D. and Reubinoff, B.E. (2008) Laser-assisted derivation of human embryonic stem cell lines from IVF embryos after preimplantation genetic diagnosis. *Hum Reprod*, **23**, 46-53.

2 Harper, J.C., Wells, D., Piyamongkol, W., Abou-Sleiman, P., Apessos, A., Ioulianos, A., Davis, M., Doshi, A., Serhal, P., Ranieri, M. *et al.* (2002) Preimplantation genetic diagnosis for single gene disorders: experience with five single gene disorders. *Prenat Diagn*, **22**, 525-533.

3 Irizarry, R.A., Hobbs, B., Collin, F., Beazer-Barclay, Y.D., Antonellis, K.J., Scherf, U. and Speed, T.P. (2003) Exploration, normalization, and summaries of high density oligonucleotide array probe level data. *Biostatistics*, **4**, 249-264.

4 Eden, E., Navon, R., Steinfeld, I., Lipson, D. and Yakhini, Z. (2009) GOrilla: a tool for discovery and visualization of enriched GO terms in ranked gene lists. *BMC Bioinformatics*, **10**, 48.

5 Franceschini, A., Szklarczyk, D., Frankild, S., Kuhn, M., Simonovic, M., Roth, A., Lin, J., Minguez, P., Bork, P., von Mering, C. *et al.* (2013) STRING v9.1: protein-protein interaction networks, with increased coverage and integration. *Nucleic Acids Res*, **41**, D808-815.

6 Elkon, R., Linhart, C., Sharan, R., Shamir, R. and Shiloh, Y. (2003) Genome-wide in silico identification of transcriptional regulators controlling the cell cycle in human cells. *Genome Res*, **13**, 773-780.

7 Shamir, R., Maron-Katz, A., Tanay, A., Linhart, C., Steinfeld, I., Sharan, R., Shiloh, Y. and Elkon, R. (2005) EXPANDER--an integrative program suite for microarray data analysis. *BMC Bioinformatics*, **6**, 232.

8 Smyth, G. K. (2004) Linear models and empirical bayes methods for assessing differential expression in microarray experiments. *Stat Appl Genet Mol Biol* **3**, Article3.

9 Lonnstedt, I. S., T. (2002) Replicated microarray data. *Statistica Sinica*, **12**, 31-46.

10 Reiner, A., D. Yekutieli and Y. Benjamini (2003).Identifying differentially expressed genes using false discovery rate controlling procedures. *Bioinformatics,* **19,** 368-375.
